# Metatranscriptomic analysis reveals dissimilarity in viral community activity between an ice-free and ice-covered winter in Lake Erie

**DOI:** 10.1128/msystems.00753-24

**Published:** 2024-06-28

**Authors:** Elizabeth R. Denison, Brittany N. Zepernick, R. Michael L. McKay, Steven W. Wilhelm

**Affiliations:** 1 Department of Microbiology, University of Tennessee, Knoxville, Tennessee, USA; 2 Great Lakes Institute for Environmental Research, University of Windsor, Windsor, Ontario, Canada; University of Massachusetts Amherst, Amherst, Massachusetts, USA

**Keywords:** viral diversity, winter limnology, metatranscriptomics, climate change, diatom bloom

## Abstract

Winter is a relatively under-studied season in freshwater ecology. The paucity of wintertime surveys has led to a lack of knowledge regarding microbial community activity during the winter in Lake Erie, a North American Great Lake. Viruses shape microbial communities and regulate biogeochemical cycles by acting as top-down controls, yet very few efforts have been made to examine active virus populations during the winter in Lake Erie. Furthermore, climate change-driven declines in seasonal ice cover have been shown to influence microbial community structure, but no studies have compared viral community activity between different ice cover conditions. We surveyed surface water metatranscriptomes for viral hallmark genes as a proxy for active virus populations and compared activity metrics between ice-covered and ice-free conditions from two sampled winters. Transcriptionally active viral communities were detected in both winters, spanning diverse phylogenetic clades of putative bacteriophage (*Caudoviricetes*), giant viruses (*Nucleocytoviricota*, or NCLDV), and RNA viruses (*Orthornavirae*). However, viral community activity metrics revealed pronounced differences between the ice-covered and ice-free winters. Viral community composition was distinct between winters and viral hallmark gene richness was reduced in the ice-covered relative to the ice-free conditions. In addition, the observed differences in viral communities correlated with microbial community activity metrics. Overall, these findings contribute to our understanding of the viral populations that are active during the winter in Lake Erie and suggest that viral community activity may be associated with ice cover extent.

**IMPORTANCE:**

As seasonal ice cover is projected to become increasingly rare on large temperate lakes, there is a need to understand how microbial communities might respond to changing ice conditions. Although it is widely recognized that viruses impact microbial community structure and function, there is little known regarding wintertime viral activity or the relationship between viral activity and ice cover extent. Our metatranscriptomic analyses indicated that viruses were transcriptionally active in the winter surface waters of Lake Erie. These findings also expanded the known diversity of viral lineages in the Great Lakes. Notably, viral community activity metrics were significantly different between the two sampled winters. The pronounced differences we observed in active viral communities between the ice-covered and ice-free samples merit further research regarding how viral communities will function in future, potentially ice-free, freshwater systems.

## INTRODUCTION

Decades of research have demonstrated that viruses are key biological components of aquatic ecosystems (1). Through top-down control, viruses influence microbial community structure and the movement of organic matter *via* the “viral shunt” and “viral shuttle” (2–4). Viruses also modulate the metabolism of infected cells by hijacking host cell physiology and through virus-encoded metabolic genes (5), which can have far-reaching implications for biogeochemical cycles (6, 7). As many of Earth’s aquatic ecosystems are faced with the unprecedent changes in climate (8, 9), there is growing interest in how environmentally relevant viruses are influenced by climate change, such as how climate modulates viral diversity and production rates (10). The winter period in temperate freshwater lakes is particularly vulnerable to climate change, where one apparent response is the decline in ice cover duration and thickness (9, 11). In fact, the frequency of ice-free winters is projected to increase globally in current climate trajectories (12). Seasonally ice-covered lakes are not biologically dormant during the winter (13), and yet there is relatively little documented about viral activity during this period (14).

The Great Lakes contain about 20% of the global freshwater supply (15) and have substantial contributions to regional socioeconomic services (16). Winter in the Laurentian Great Lakes is undergoing pronounced changes due to a warming climate (17). Since the 1970s, Lake Erie has exhibited significant declines in wintertime ice cover extent and duration (18, 19). Furthermore, ice cover extent drastically alters microbial community structure by shifting the winter ecosystem from one characterized by dense accumulations of filamentous diatoms (20–22) to one dominated by picoplankton (23). Putative phage gene fragments (24) and RNA virus sequences (25) have been detected during the mid-winter in Lake Erie, but there has been no comprehensive characterization of winter viral activity in the Great Lakes to date. In addition, the relationship between viral community activity and ice cover extent has not previously been explored. Viral community composition and activity are shaped by the surrounding microbial community (26, 27). Given that ice cover can influence microbial population structure, it is reasonable to expect a relationship between ice cover extent and viral activity. Viral activity could also be directly impacted by other ice cover-dependent factors as well (e.g., the effect of UV exposure on viral decay rates (28, 29)). Characterizing active viral populations during the winter is an important step toward understanding how virus-mediated processes may be impacted by changing ice conditions.

Targeted gene amplification and shotgun metagenomic approaches have been informative in the study of viruses in the Great Lakes (24, 30, 31), but a limitation of DNA-based approaches is that they do not reveal virus activity (i.e., replicating viruses) or capture RNA viruses. Metatranscriptomics can facilitate both the discovery of RNA viruses and estimate *in situ* activity levels of viruses using transcript abundance as a proxy (32). Here, we surveyed metatranscriptomes from filtered (>0.2 µm) samples collected from Lake Erie surface waters during two contrasting winters: one winter with high-ice cover (2019, 95% mean maximum ice cover) and a subsequent relatively ice-free winter (2020, 15% mean maximum ice cover) (33). Samples from the spring months following the ice-free winter were also collected and served as an outgroup in this study. Using hallmark genes as proxies for putative viral populations, we identified phylogenetically diverse viral communities that were transcriptionally active in the winter surface waters. Viral community activity metrics (alpha and beta diversity indices) revealed significant differences in community composition and viral richness (i.e., the number of different viral hallmark gene variants) between the ice-covered and ice-free samples. In addition, viral activity metrics were correlated with microbial community activity metrics, namely with microbial richness and the proportion of diatom reads within the winter libraries. This suggested that the differences in viral activity between the winters may in part be linked to differences in the surrounding microbial community. Although the cause(s) of the observed differences in viral community activity remain uncertain, they coincided with the substantial variation in ice cover extent between the winters.

## MATERIALS AND METHODS

### Sample collection

Opportunistic surface water samples were collected by USCGC *Neah Bay* between February and March of two consecutive years (2019 and 2020). Additional spring samples in 2020 were collected by MV *Orange Apex*. Samples were collected from 0.5 meters below the surface as either whole water (*n* = 20) or net concentrated (*n* = 57, 64 µm net opening). Samples intended for RNA extraction were passed through 0.22 µm nominal pore-size filters, flash-frozen, and stored at −80°C until further processing. Details regarding RNA extraction and sequence processing can be found in an associated *Microbiology Resource Announcement* (34). Meteorological and surface water conditions, ice thickness, nutrient concentration, chlorophyll *a* (>20 µm and >0.22 µm size classes), phytoplankton taxonomy, and phytoplankton cell abundance were recorded. Chlorophyll *a* measurements were performed using the method described by Twiss et al. (21). Nutrient measurements were performed by the National Center for Water Quality Research (Heidelberg University, Tiffin, OH, USA). Phytoplankton identification and enumeration were performed as described in Zepernick et al. (35). Sample metadata can be accessed through the Biological and Chemical Oceanography Data Management Office (BCO-DMO) under data set number 809945.

### Metatranscriptome assembly and gene prediction

This study used the metatranscriptome co-assembly and gene predictions generated by Zepernick et al. (35). Briefly, in Zepernick et al., quality-filtered reads were co-assembled using MEGAHIT v1.2.9 (https://github.com/voutcn/megahit) and the resulting co-assembly was assessed using QUAST v5.0.2 (Table S1) (https://github.com/ablab/quast). Gene sequences were predicted from the co-assembly contigs using MetaGeneMark v3.38 (36). Here, to detect and quantify the relative transcript abundance for genes within a sample, we mapped trimmed reads to gene sequences with ≥90% identity and ≥90% read length fraction thresholds using CoverM v0.6.1 (https://github.com/wwood/CoverM). To remove poorly covered genes, at least 50% of the gene must be covered to be considered detected in a metatranscriptome library (37). Read counts for genes with less than 50% coverage were reset to zero on a per-library basis. Read counts were normalized using the transcripts per million (TPM) method (38).

### Viral hallmark gene discovery

Conserved viral hallmark genes were identified as proxies for viral populations to estimate viral diversity and activity, similar to as performed previously (32, 39). All hallmark genes were identified through protein sequence similarity searches. Predicted protein sequences from MetaGeneMark were aligned to databases of viral hallmark genes (detailed below) using DIAMOND BLASTp (https://github.com/bbuchfink/diamond) (E-value threshold of 1e^−5^). Protein sequences that aligned to translated hallmark genes were then aligned to the RefSeq v213 database and sequences with a top hit to a viral protein were retained as putatively viral (32). As a second quality control measure, only viral sequences with an appropriate protein domain identified by a Pfam domain search (database v32) were retained for analysis (i.e.*,* a putative Gp23 sequence retrieved from the BLASTp that had no capsid domain was not included). RdRp-like protein sequences were further filtered using Palmscan (-rdrp option) and only high confidence RdRp sequences retained (https://github.com/rcedgar/palmscan). As an additional measure to remove non-viral sequences, the metatranscriptome contig from which the hallmark gene originated was piped through VirSorter2 v2.2.3 (https://github.com/jiarong/VirSorter2) and CheckV v1.0.1 (https://bitbucket.org/berkeleylab/CheckV). While the majority of contigs were too short to determine whether they were non-viral, no hallmark gene contigs had any cellular genes called by VirSorter2 or CheckV.

### Hallmark gene databases

The terminase large subunit (TerL) was used as a general phage marker (i.e.*,* a marker that is present in all *Caudoviricetes*) (Table S2) (40). Relatively few TerL were detected, most of which resembled T4-like myophage based on sequence homology. Therefore, the T4-like major capsid protein (Gp23), DNA polymerase B (Gp43), and tail sheath domains were used to detect myophage. The Gp23 had the greatest richness compared to the other phage markers and was therefore chosen as the representative hallmark gene for myophage for further analysis (Table S2). Viral integrase, excisionase, CI repressor, and Cro repressor were also screened for as these markers have been used to detect lysogenic phage in metagenomic data (41, 42). All *Caudoviricetes* marker sequences were retrieved from Interpro, where only viral sequences were downloaded.

The major capsid protein (MCP) was used as a proxy for active *Nucleocytoviricota*, or NCLDV, populations. We chose the MCP marker because (i) it is conserved in many NCLDV genomes (43) (all known groups except for *Pandoraviridae* and *Pithoviridae*) and (ii) it is most likely to be detectable since the MCP can be highly expressed relative to other NCLDV core genes (44). We also searched for DNA polymerase B (PolB) as an alternative NCLDV marker, but only nine PolB sequences were detected in the metatranscriptomes *via* read mapping (Table S2). Therefore, we only discuss the MCP as a proxy for NCLDV with the caveat that the MCP may not be the most robust phylogenetic marker for NCLDV (45). NCLDV marker sequences were retrieved from the NCVOG database (https://ftp.ncbi.nih.gov/pub/wolf/COGs/NCVOG/).

RNA-dependent RNA polymerase (RdRp) was used as the hallmark gene for RNA viruses (*Orthornavirae*) (46), and the RdRp-Scan database was used for RdRp searches (https://github.com/JustineCharon/RdRp-scan). Replicase sequences compiled by Kazlauskas et al. (47) and Moniruzzaman et al. (32) were used to screen for circular replicase-encoding single-stranded (CRESS) DNA viruses (*Cressdnaviricota*).

### Phylogenetic analysis

Hallmark protein sequences were aligned to reference alignments using Clustal Omega v1.2.4 (76). Positions with >90% gaps were trimmed from the alignments by trimAl v1.4 (https://github.com/inab/trimal). Trees were constructed using FastTree v2.1.11 (-lg option) (http://www.microbesonline.org/fasttree) or IQ-TREE v2.2.0.3 (-m TEST option) (http://www.iqtree.org) and were visualized in iTOL (80). NCLDV reference genomes were retrieved from Gilbert et al. (81) and the MCP sequences were predicted using ViralRecall v2 (https://github.com/faylward/viralrecall). RdRp-Scan alignments were used for the RdRp tree references. References for all other trees were manually curated.

### Microbial community characterization

Taxonomic annotation and functional annotation of predicted cellular genes were retrieved from Zepernick et al*.* (35). Briefly, taxonomy was estimated using EUKulele v1.0.6 (https://github.com/AlexanderLabWHOI/EUKulele), where protein sequences were aligned to the PhyloDB database v1.076 by DIAMOND BLASTp. Cellular genes were functionally annotated by eggNOG-mapper v2.1.6 using the eggNOG 5.0 orthology database with a DIAMOND BLASTp cutoff of 1e^−10^ (https://github.com/eggnogdb/eggnog-mapper).

### Statistical analyses

Community diversity metrics were calculated using the R package vegan (https://github.com/vegandevs/vegan) and in PRIMER v7 (48, 49). Normalized transcript abundance (TPM) tables were used as the input for all diversity analyses. For beta diversity statistics, Bray-Curtis dissimilarity matrices were generated from the standardized TPM table (Wisconsin double standardization). Similarity percentage (SIMPER) analyses were used to quantify the contribution of individual marker genes to average dissimilarity between sample groups.

Spearman’s correlations were performed using R (cor.test) (48). The virus-host hallmark gene interaction network was generated using FlashWeave (*α*  < 0.01) (https://github.com/meringlab/FlashWeave.jl), where genes annotated as DNA-directed RNA polymerase subunits Rpb1 (KEGG orthology K03006) and RpoB (K03043) were used as cellular hallmark genes (32, 50). A TPM table of all viral and cellular hallmark genes was used as input for the correlation analysis.

## RESULTS

### Preliminary comparison of viral community activity between whole-water and net-concentrated samples

Beta diversity analysis based on the relative transcript abundance of viral hallmark genes showed that the composition of active viral communities was significantly different between the whole-water (no filtration) and net-concentrated (64 µm opening) samples (ANOSIM R = 0.91, *P* = 0.001) (Fig. S1A). The total number of putative viral hallmark gene variants detected within a library was significantly lower in the net-concentrated relative to the whole-water samples (ANOVA, *P* < 0.001) (Fig. S1B). Examination of the relative abundance of viral hallmark genes within the libraries revealed that phage (Gp23), NCLDV (MCP), and RNA virus (RdRp) transcripts were detected in each whole-water sample, but that phage and NCLDV transcripts were relatively absent in the net-concentrated metatranscriptomes (Fig. S1C). Since we observed more hallmark gene variants in the unfiltered samples, both in terms of the number of hallmark genes detected and the different virus types detected (phage, NCLDV, and RNA viruses), we focused on the whole-water samples and the net-concentrated metatranscriptomes are not discussed here further.

### Physiochemical parameters and phytoplankton abundance estimates

The whole-water samples spanned temporal, climatic, and spatial gradients. Whole-water samples were collected during February–March of 2019 (*n* = 4), February–March of 2020 (*n* = 10), and May–June of 2020 (*n* = 6) across 12 sites (Fig. S2; Table S3). The winter of 2019 was considered a high-ice year (95% mean maximum ice cover) while the winter of 2020 was largely ice-free (15% mean maximum ice cover) (33). Ice cover ranged from 90% to 100% at the 2019 winter (i.e., February/March) sample sites and was 0% at all 2020 winter sample sites (Table S3). Ice thickness ranged from approximately 2.5 to 15 cm at the ice-covered sites. The concentration of dissolved ammonia, chlorine, silicate, and soluble reactive phosphorus and the silica:nitrate ratio were not significantly different between 2019 (ice-covered) and 2020 (ice-free) winters (two-tailed unpaired *t*-test, *P* > 0.1) (Fig. S3; Table S4). Dissolved nitrate was on average significantly higher in the ice-covered relative to the ice-free winter (*P* = 0.03) (Fig. S3). Chlorophyll *a* (both >20 µm and >0.22 µm size classes) and diatom cell abundance were on average lower in the ice-free samples, but not significantly so (*P* > 0.1) (Fig. S4; Table S5). An in-depth analysis on phytoplankton dynamics is outside of the scope of this study, but details regarding phytoplankton taxonomy and distribution in these samples can be found in the associated study by Zepernick et al. (35).

### Signatures of active viral communities within the winter surface water metatranscriptomes

Viral hallmark genes detected in the metatranscriptomes were interpreted to represent transcriptionally active or cell-associated viral populations. This was presumed because (i) DNA viruses must be replicating intracellularly to synthesize mRNA and (ii) samples were filtered (>0.2 µm) prior to nucleic acid extraction at a size cutoff that would enrich for cell-associated RNA viruses. Hereafter we refer to the detected virus populations as “active” with the consideration that this approach may detect cell-associated RNA virus genomes that are not replicating.

**Fig 1:**
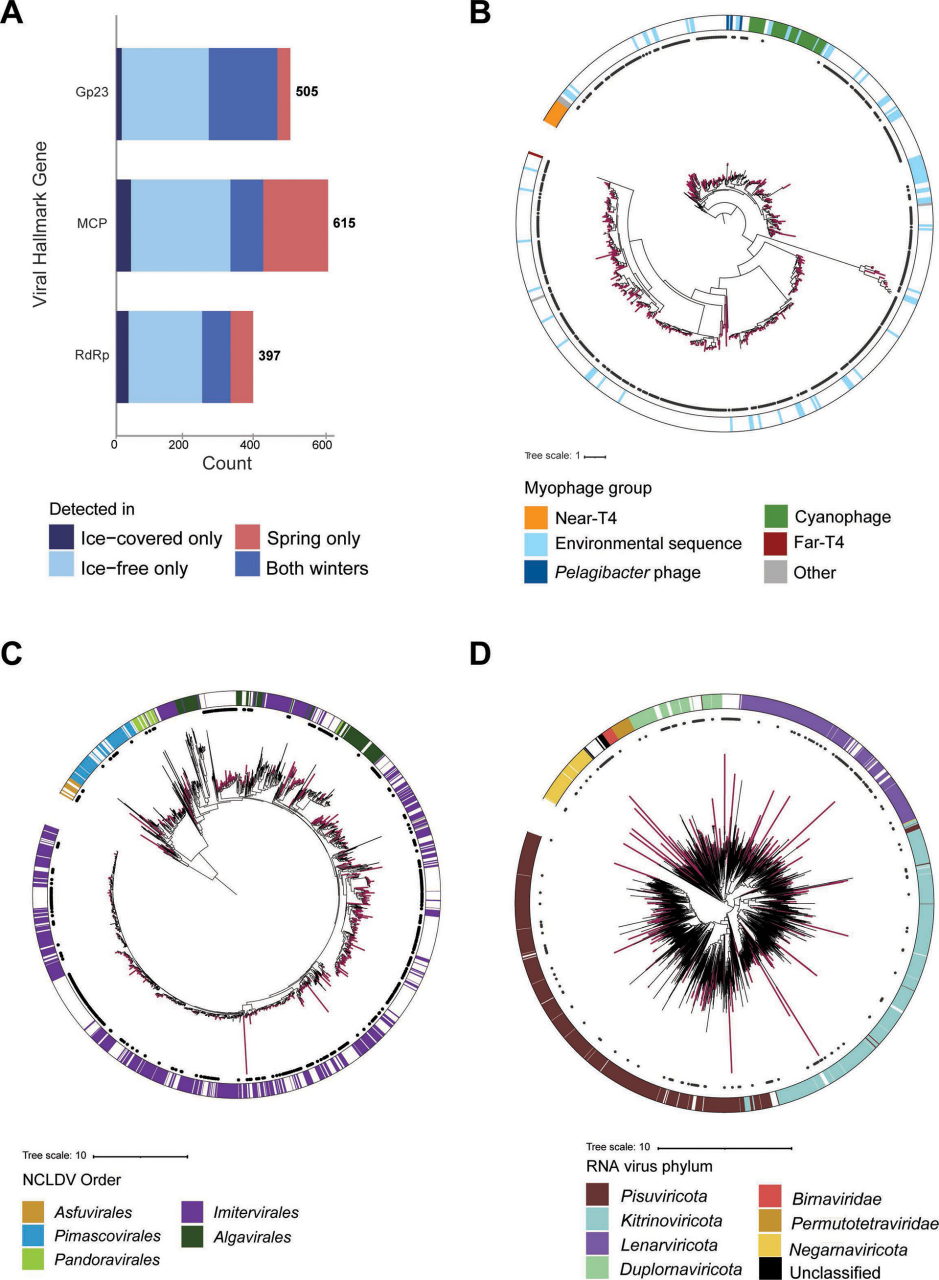
Viral hallmark genes detected in the surface water metatranscriptomes. (**A**) Total count of genes discovered for Gp23, MCP, and RdRp. Maximum likelihood trees for (B) Gp23, (**C**) MCP, and (D) RdRp. Branches for Lake Erie sequences are bolded and in color, reference sequence branches are in black. Inner ring circles represent Lake Erie sequences detected in at least one winter library. The outer ring shows the taxonomy of the reference sequence.

Putative phage (Gp23), NCLDV (MCP), and RNA virus (RdRp) hallmark genes were detected in the winter surface water samples (Fig. 1A). Overall, active winter viral communities displayed high hallmark gene richness (i.e., the number of hallmark gene variants detected) and were phylogenetically diverse (Fig. 1A through D). The majority of Gp23 detected in the winter metatranscriptomes *via* read mapping (439 predicted gene sequences) were most closely related to uncultured myophage environmental sequences with uncertain hosts as opposed to the established cultured clades, although some Gp23 were related to isolated cyanophage (two sequences) and *Pelagibacter* phage (six sequences) (Fig. 1B; Table S6). The NCLDV MCP detected in the winter libraries (427 sequences) represented five of the six established NCLDV orders (Fig. 1C) (71), most of which were assigned to the *Imitervirales* (395 sequences) (Table S6). The relatively higher *Imitervirales* MCP richness may reflect the broad host range of this group (92, 93). The phylogenetic diversity of RdRp present in the winter libraries (332 sequences) was similarly high, spanning the known phylum-level diversity of the *Orthornavirae* (Fig. 1D; Table S6). Furthermore, only a single lysogenic marker (integrase) was considered viral based on its top BLASTp hit to uncultured freshwater *Caudoviricetes* (GenBank accession: CAB5220699.1) and was only present in one library in the ice-free conditions (MT6, 02/14/2020).

The viral hallmark gene transcript pool was consistently dominated by Gp23 and RdRp transcripts across both winters, although MCP representation increased in the spring, specifically the May 1–22 samples (Fig. S5). Only eight single-stranded DNA virus replicase markers were detected with low representation in the water column libraries (Table S2; Fig. S5) and they were not analyzed further.

### Viral community activity metrics differed between the ice-covered and ice-free conditions

**Fig 2:**
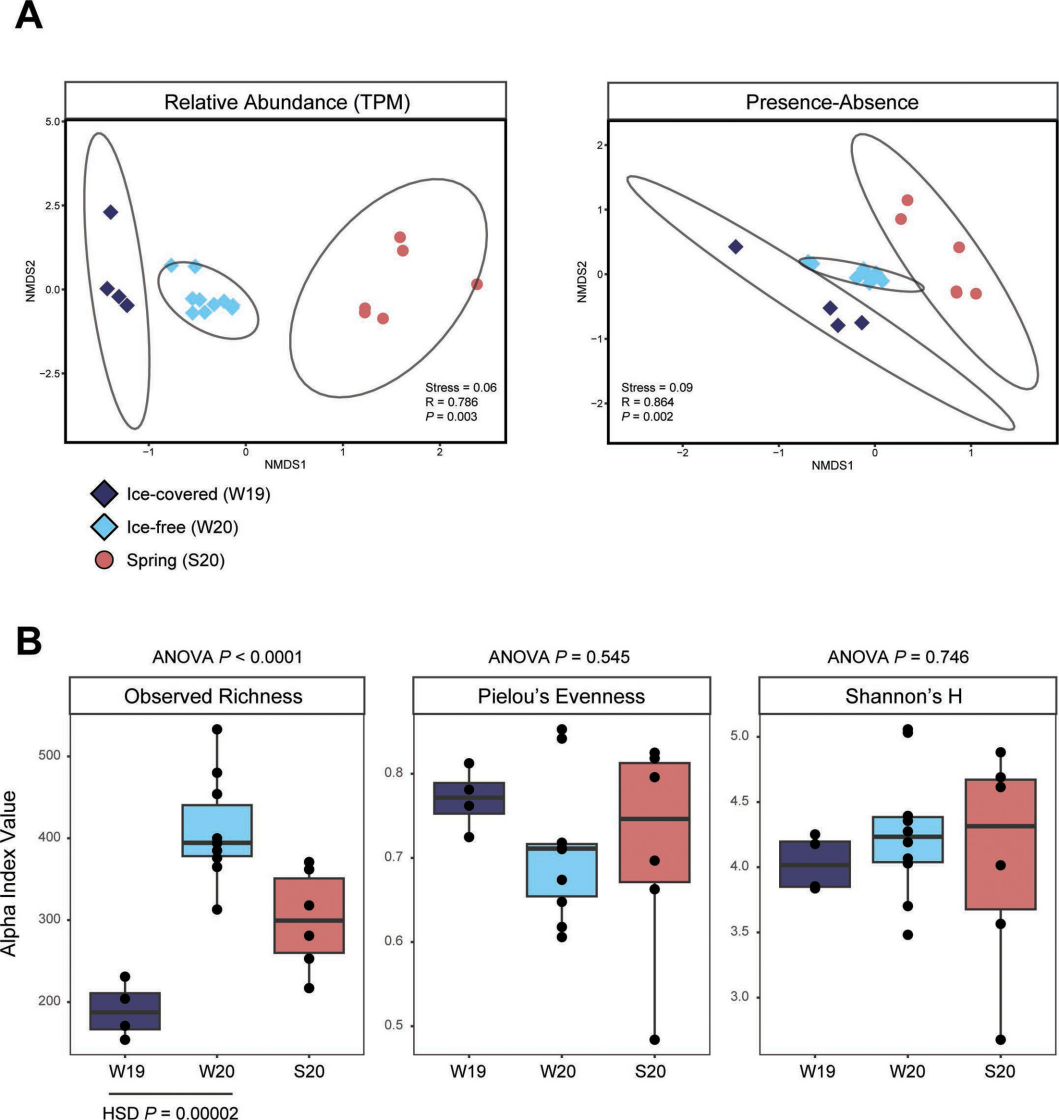
Comparison of viral activity metrics between the two winters. Beta diversity non-metric multidimensional scaling (nMDS) plot illustrating the similarity of a sample structure is based on (A) relative transcript abundance and (B) presence-absence transformed abundance of viral markers. ANOSIM results are shown for the pairwise winter comparison. Ellipses represent 95% confidence intervals for the three seasons sampled. (**C**) Alpha diversity metrics grouped by season. Tukey HSD is shown for the ice-covered winter 2019 (**W19**) and ice-free winter 2020 (**W20**) sample comparison when applicable.

Alpha and beta diversity metrics were calculated based on the detected viral hallmark genes to compare viral community activity between the two winters. Community activity was highly dissimilar between the ice-covered and ice-free conditions when comparing the relative abundance (TPM) of viral hallmark genes (73% dissimilar on average, SIMPER analysis). Furthermore, non-metric multidimensional scaling (nMDS) plots revealed that viral community composition clustered by season and that composition was significantly different between the winters based on both hallmark gene relative abundance (ANOSIM R = 0.79, *P* = 0.003) and presence-absence matrices (ANOSIM R = 0.86, *P* = 0.002) (Fig. 2A). Mean viral community evenness (Pielou’s) and diversity (Shannon’s H) were not significantly different between seasons (Fig. 2B; Table S7). While community evenness was high in all winter samples (Pielou’s J > 0.6), evenness was on average lower in the ice-free samples. Mean hallmark richness, however, was significantly lower (Tukey HSD *P* < 0.001) in the ice-covered condition compared to the ice-free (Fig. 2B). In other words, there were significantly fewer viral hallmark genes detected *via* read mapping in the ice-covered winter samples. Viral hallmark richness was still significantly lower when differences in sequencing depth were accounted for (i.e., when hallmark gene counts were normalized to library size) (Tukey HSD *P* < 0.001).

Viral community activity was significantly different between winters when examining virus hallmark types individually. The separate Gp23, MCP, and RdRp communities each clustered by season and were significantly different between the ice-covered and ice-free winters based on relative abundance (Fig. S6). Similarly, the mean hallmark gene richness of all three virus types was significantly lower in the ice-covered winter relative to the ice free (Fig. S7; Table S8). Overall, active viral communities displayed pronounced differences in diversity when compared between the ice-covered and ice-free winter samples and these differences were observed in a broad range of virus types.

### Differences in viral community activity were correlated with microbial community activity metrics

**Fig 3:**
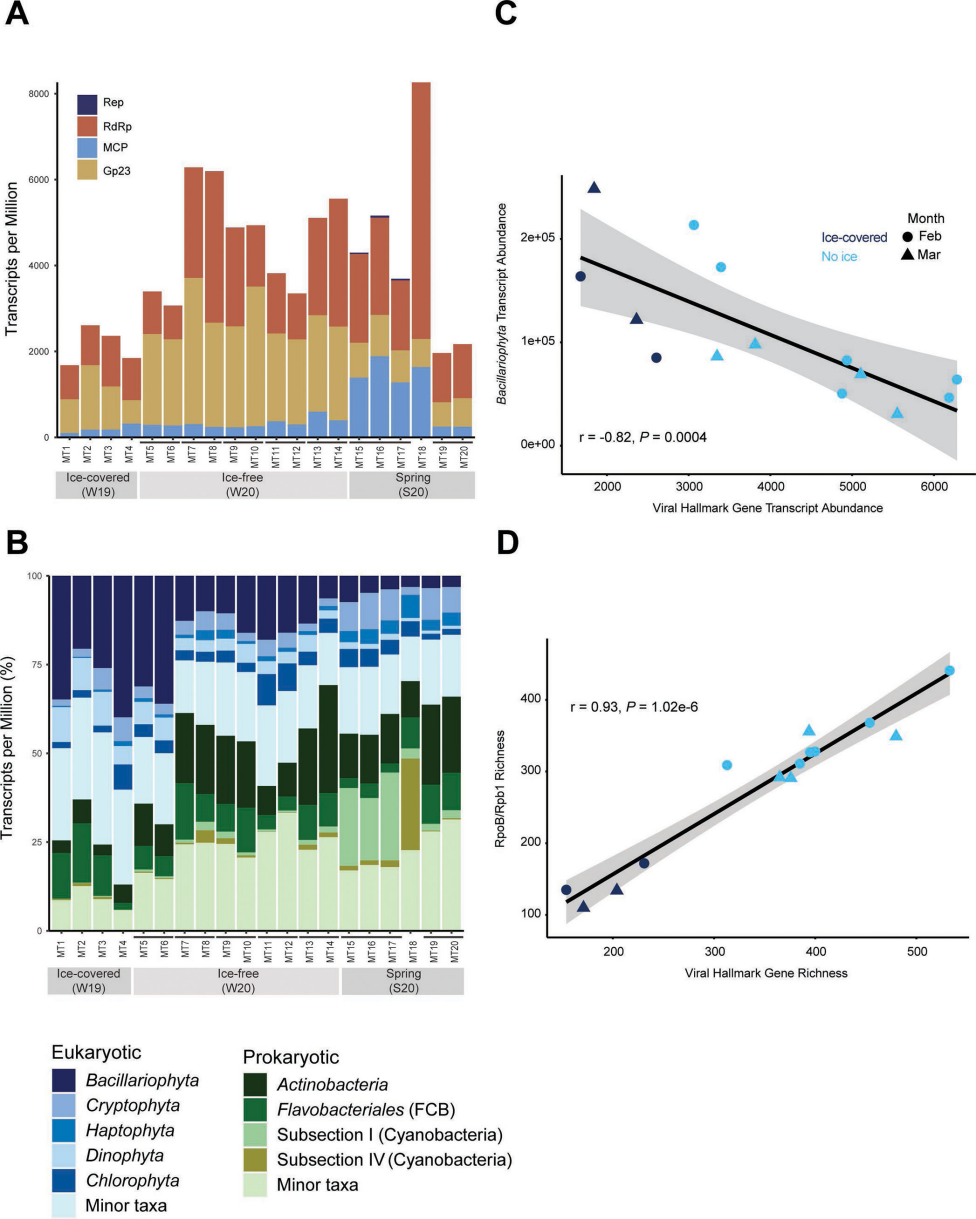
**(A**) Relative transcript abundance (TPM) of the viral hallmark genes myophage (Gp23), NCLDV (MCP), RNA viruses (RdRp), and CRESS DNA viruses (Rep). (**B**) Relative transcript abundance of the dominant eukaryotic and prokaryotic taxa. Spearman’s correlations between (C) viral hallmark and diatom (*Bacillariophyta*) transcript abundance and (D) viral and cellular hallmark gene richness. Biological replicates are connected by horizontal bars on the bar charts on panels A and B.

Collectively, viral hallmark genes comprised a larger portion of the transcript pool (i.e.*,* total TPM) in the ice-free samples compared to the ice-covered (Fig. 3A). Higher relative viral transcript abundance in the ice-free conditions coincided with a lower abundance of the bloom-forming diatoms (*Bacillariophyta*) in terms of both diatom relative transcript abundance (Fig. 3B) and cell counts (Fig. S4). Conversely, prokaryotic transcripts had higher representation in the ice-free conditions (13%–36% versus 30%–67%, respectively) (Fig. 3B). In fact, collective prokaryotic relative abundance was significantly negatively correlated with diatom relative abundance across the winter samples (Spearman’s ρ = −0.83, *P* < 0.001) (Table S9). Moreover, both prokaryotic (*rpoB*) (Tukey HSD *P* < 0.001) and eukaryotic (*rpb1*) (Tukey HSD *P* = 0.02) hallmark gene richness were lower in the ice-covered winter (Fig. S8).

The shifts in microbial community metrics correlated with the observed differences in viral hallmark gene abundance and richness. Viral hallmark gene relative transcript abundance was significantly negatively correlated with diatom relative transcript abundance (ρ = −0.82, *P* < 0.001) (Fig. 3C) and positively so with prokaryotic abundance (Table S9). Furthermore, viral richness was positively correlated with microbial community richness (RpoB/Rpb1) (ρ = 0.93, *P* = 2.2e-16) (Fig. 3D). Altogether, lower diatom representation in the ice-free metatranscriptomes was associated with higher microbial community richness and prokaryotic representation, and, in turn with higher viral richness and representation.

### Hallmark genes with the largest contributions to the dissimilarity between winter conditions

**Fig 4:**
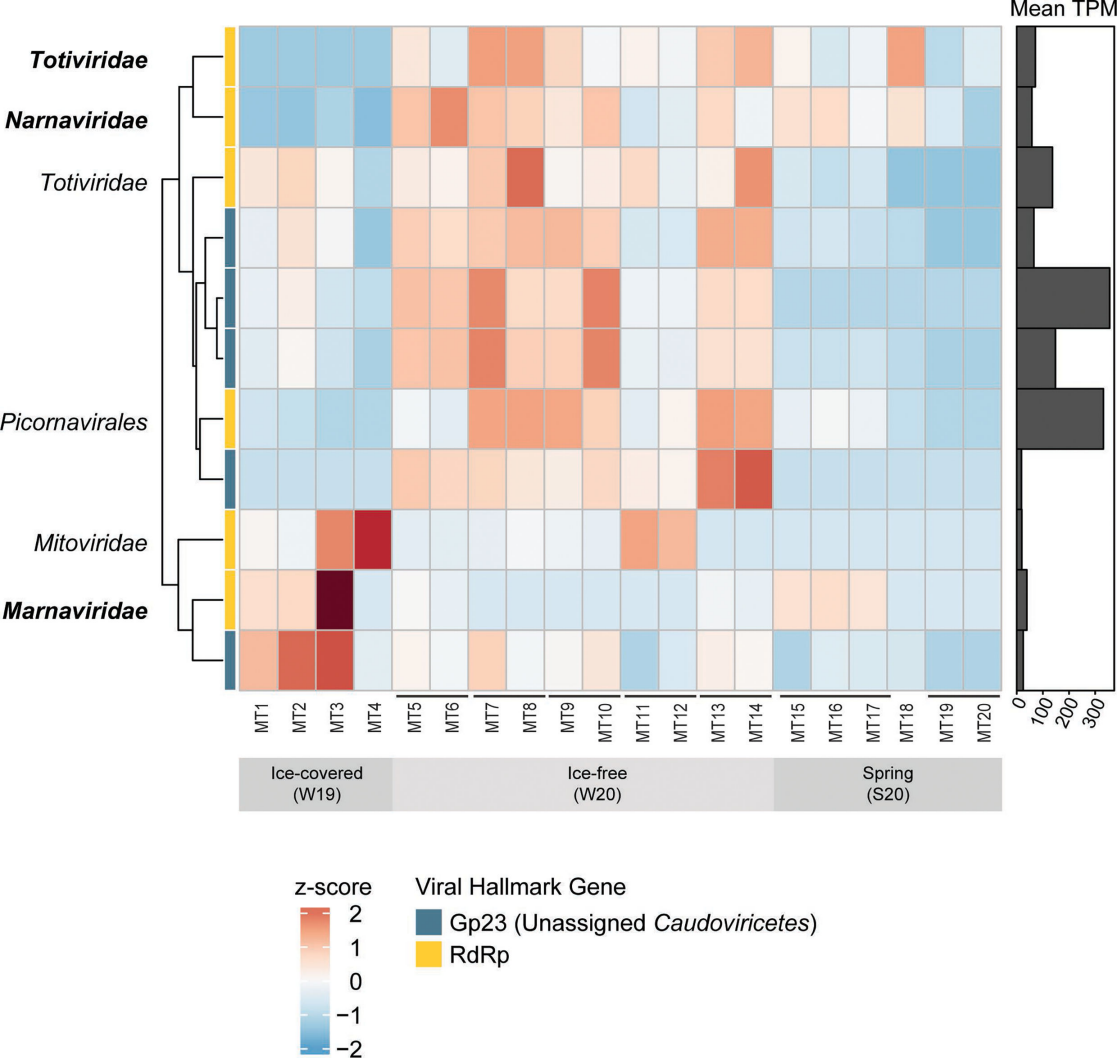
Relative transcript abundance (z-scored TPM) of the 11 top contributors to the dissimilarity between the ice-covered and ice-free samples based on SIMPER analysis. Rows are clustered by abundance pattern. The bar plot shows the mean TPM across all 20 samples. Biological replicates are connected by horizontal bars. Phylogenetic placement for RdRp are indicated by text labels, and diatom-associated RdRp clades are in bold.

Although viral richness and representation (collective TPM) were higher in the ice-free conditions, only a few individual viral markers increased in relative abundance and contributed to the dissimilarity between the ice cover conditions. Out of the total 1,525 viral hallmark genes, only 11 had greater than 0.5% contribution to average dissimilarity and amounted to the cumulative 10% average dissimilarity between the ice cover conditions based on the SIMPER analysis (Table S10). While this cut-off is somewhat arbitrary, it captures the viral markers with the largest shifts in relative abundance between the winters, and the remaining markers have low individual contributions. Eight of the eleven markers of interest (four Gp23 and four RdRp) had higher average representation in the ice-free relative to the ice-covered winter where they “spiked” in relative transcript abundance in the ice-free conditions, while the remaining three (one Gp23 and two RdRp) had the opposite trend (Fig. 4). Notably, the three markers that had higher average representation in the ice-covered winter had an overall low relative abundance (Fig. 4), which is in line with the overall low viral TPM in the ice-covered winter samples noted previously.

All five of the top Gp23 contributors were placed within a clade containing an uncultured freshwater Gp23 sequence with an uncertain host range (GenBank accession CAB5226458.1). The RdRp of interest spanned three RNA virus phyla (Fig. 4). Interestingly, three RdRp markers were related to diatom viruses within the *Bacillarnavirus* (order *Picornavirales*) (Fig. S9) and diatom colony-associated viruses within the *Narnaviridae* (phylum *Lenarviricota*) (Fig. S10) and *Totiviridae* (phylum *Duplornaviricota*) (Fig. S11). To further infer virus-host pairs, we assessed correlations in relative transcript abundance between the cellular marker genes (*rpoB*/*rpb1*) and the virus hallmark genes, but no significant interactions (*α*  < 0.01) were found between the diatom Rpb1 and putative diatom virus RdRp markers.

Major capsid protein transcript abundance was consistently low throughout both winters, and subsequently, no single MCP marker had a large contribution to the dissimilarity between winters. However, MCP had higher representation in the spring samples (May 1–22) relative to both winters largely due to the relative increase in two MCPs. Their dominance was also likely responsible for the lower evenness within the MCP communities in the spring samples (Fig. S7). Although host range is uncertain, these MCPs resembled isolated Chloroviruses (order *Algavirales*) that infect green algae and *Mimiviridae* (order *Imitervirales*) isolates that infect amoeba (Fig. 1C). It was noteworthy that the Chlorovirus-like MCP had a significant positive correlation with a Rpb1 annotated as Chlorophyta (*Trebouxiophyceae*) (*α*  < 0.01, cor >0.8).

## DISCUSSION

Viral activity during the winter months in the Great Lakes is largely undocumented. Here, viral hallmark genes were detected in metatranscriptomes from two contrasting winters in Lake Erie, indicating that there are viral communities “active” in the surface waters. Our findings expanded the known diversity of viruses that have been detected during the winter in Lake Erie. In one of the few studies to quantify viruses in winter samples collected from the Great Lakes, Matteson et al. enumerated putative cyanophage in mid-winter samples and found them to be a substantial component of the standing stock of viruses (24). However, the qPCR-based detection approach that was used only characterized a narrow group of viruses (cyanomyophage of *Synechococcus* spp.) and could not reveal if those viruses were transcriptionally active. While our metatranscriptomic approach also detected cyanomyophage-like hallmark genes, they represented a small component of the total myophage phylogenetic diversity. Instead, most myophage markers detected here belonged to clades of putative aquatic viruses with unknown hosts. It was previously also hypothesized that phage may “overwinter” as prophage in the Lake Erie water column (24). While we do not dismiss lysogeny as a component of Lake Erie winter microbial communities, we detected little evidence of lysogenic lifestyles based on the transcripts assembled here. Metagenomic sequencing may enable the assembly of phage genomes that could help clarify the prevalence of temperate phage in Lake Erie.

Diverse populations of putative eukaryotic viruses within the NCLDV and RNA virus groups were also discovered in the winter samples. Previously, only 11 dsRNA viruses within the *Partitiviridae* (*Pisuviricota*), *Totiviridae* (*Duplornaviricota*), and *Birnaviridae* (*incertae sedis*) families were identified in a single Lake Erie metatranscriptome by Edgar et al. (25), whereas here we detected hundreds of putative RNA viruses belonging to all five currently established phyla. While perhaps a limitation of sequencing depth and sample size, Edgar et al. detected no NCLDV transcripts using a similar hallmark gene approach (25). Our survey, however, revealed putative NCLDV spanning most of the established diversity (five of the six NCLDV orders). Regarding viral discovery, one consideration when using a hallmark gene approach is that viral sequence detection is limited by the diversity of the database (i.e., highly divergent viruses may not be detected by sequence homology searches). In addition, metatranscriptomes represent “snapshots” of the environment at the time and conditions sampled; therefore, the true diversity of active viruses in the surface waters was likely not captured.

Based on the putative viral sequences we identified, active virus communities were compositionally distinct between winters and viral richness was significantly lower in the ice-covered winter. Our findings showed that the observed differences in active viral communities strongly correlated with the shifts in the microbial community, suggesting a direct relationship between active viral and microbial community composition. Similar to previous observations (23), we saw evidence of a decrease in the magnitude of the winter diatom bloom in the ice-free conditions compared to the ice-covered state determined by both the representation in the metatranscriptomes (diatom relative transcript abundance) and cell count data. Other studies in aquatic environments have attributed lower viral abundance or richness in the winter to lower potential host pool abundance (51, 52), and, in principle, viral diversity and abundance are shaped by the surrounding susceptible host diversity and density (53). The drivers behind the observed differences in viral activity between winters are uncertain, but perhaps the decrease in diatom representation and increase in microbial richness contributed to the greater viral richness in the ice-free winter metatranscriptomes.

The higher levels of viral activity (viral TPM) in the ice-free winter relative to the ice-covered winter were primarily due to increased representation of a few hallmark gene variants. In other words, most viral hallmark genes did not display higher relative transcript abundance in ice-free conditions and were instead consistently low. Interestingly, the subset of viral hallmark genes that did display higher relative transcript abundance contained hallmark genes similar to those of diatom-associated RNA viruses. This included one RdRp related to lytic *Bacillarnavirus* (order *Marnaviridae*) isolates that infect marine diatoms (54), as well as RNA viruses associated with an environmental diatom colony (55). Culture-dependent studies have shown titers of some diatom viruses increased with the proliferation of diatom blooms and have been speculated to control the bloom crash (56), but also coexist during the bloom course (57). None of the putative diatom RNA viruses interacted with diatom (or any) Rpb1 markers based on our abundance-based network analysis; thus, the hosts of these putative RNA viruses remain uncertain.

We note that NCLDV did not have large contributions to the differences in active viral community composition between ice-cover states and that overall NCLDV displayed relatively low transcript abundance during both winters. Low counts (gene copy number) of various algal viruses (*Algavirales*) have been detected during the wintertime in previous studies surveying the Great Lakes (58, 59). Our findings support the idea that NCLDV activity is generally low during winter months in the Great Lakes, but continued winter surveys are needed to resolve this.

In all, we show that diverse viral populations were an active component of the winter surface waters in Lake Erie, emphasizing the need for any future winter surveys to account for viruses when investigating microbial dynamics during the winter. While we documented distinctions in viral activity between the ice-covered and ice-free winter samples, we cannot definitively attribute those differences to ice cover extent. However, we posit that the stark difference in ice cover extent (0% versus 90%–100% cover) is likely a contributing factor to the variation in microbial and viral activity since ice cover is considered a “master variable” due to its influence on biological, biogeochemical, and physical processes in freshwater lakes (17). Aspects such as the reduced active virus richness observed in the ice-covered samples could be characteristic of ice-covered waters in Lake Erie, but ultimately further winter sampling efforts spanning temporal and climatic gradients are needed to resolve trends in viral activity associated with ice cover extent.

## Data Availability

Raw and quality-filtered reads are publicly available through the JGI Genomes Online Database (GOLD) under GOLD Study ID Gs0142002.

## SUPPLEMENTAL MATERIAL

Supplemental Materials msystems.00753-24-s0001.pdf Supplemental figures and tables.

ASM does not own the copyrights to Supplemental Material that may be linked to, or accessed through, an article. The authors have granted ASM a non-exclusive, world-wide license to publish the Supplemental Material files. Please contact the corresponding author directly for reuse.
