# Supplementary material for: Metatranscriptomic analysis reveals dissimilarity in viral community activity between an ice-free and ice-covered winter in Lake Erie
Source: mSystems. 2024 Jun 28;9(7):e00753-24. doi: 10.1128/msystems.00753-24 (PMC11264689; doi:10.1128/msystems.00753-24)
Supplement: Supplemental Materials — Supplemental figures and tables. [file msystems.00753-24-s0001.pdf]

SUPPLEMENTAL FIGURES FOR PUBLICATION ONLINE

Metatranscriptomic analysis reveals dissimilarity in viral community activity between an ice-free and ice-covered winter in a North American Great Lake

Elizabeth R. Denison[1], Brittany N. Zepernick[1], R. Michael L. McKay[2] and Steven W. Wilhelm[1#]

1) Department of Microbiology, University of Tennessee, Knoxville, Tennessee, USA
2) Great Lakes Institute for Environmental Research, University of Windsor, Windsor, Ontario, Canada

#Correspondence: Steven W. Wilhelm, wilhelm@utk.edu, 1-865-974-0665

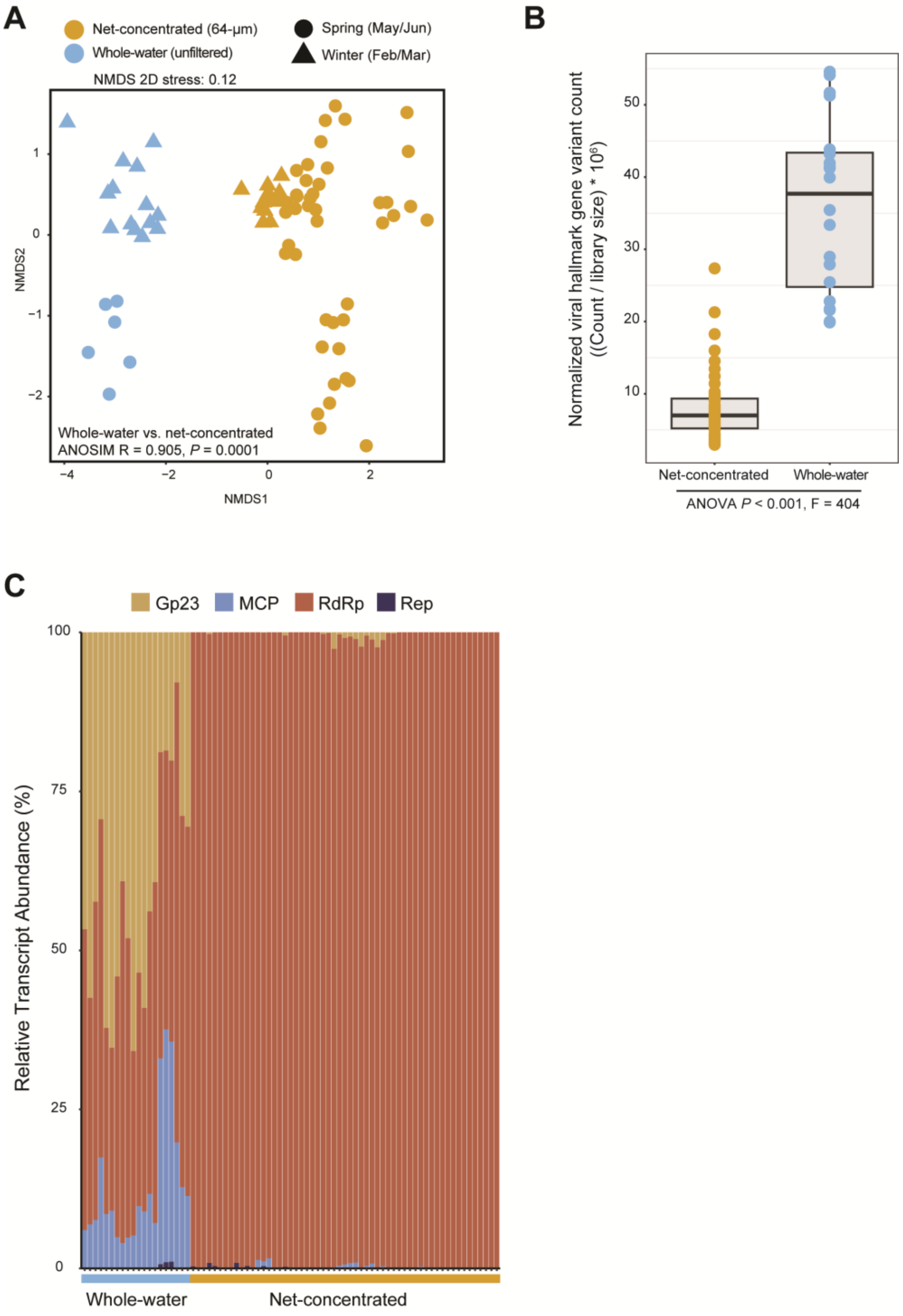

Supplemental Figure 1. A) Non-metric multidimensional scaling (nMDS) plot showing clustering of active viral communities by collection method based on the relative transcript abundance of viral hallmark genes (Bray-Curtis dissimilarity matrix). **B)** Count of viral hallmark gene variants grouped by collection method and ANOVA test results. Total counts per sample were normalized by sequencing depth and scaled. **C)** Proportion of the viral hallmark genes (Gp23, MCP, RdRp, and Rep) within each library based on summed abundance (TPM).

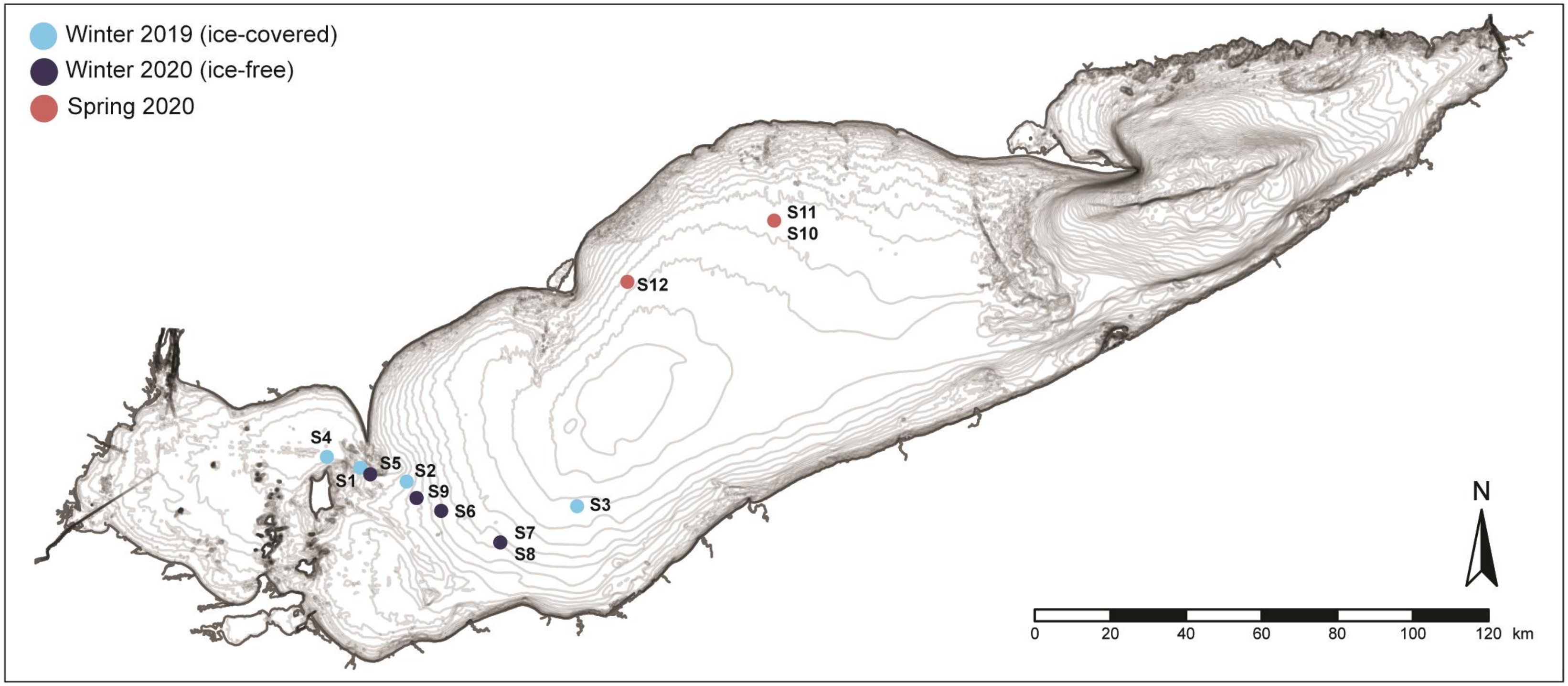

Supplemental Figure 2. Map of Lake Erie depicting the whole-water sampling sites. Site color represents the sampling period (winter 2019, winter 2020, or spring 2020). Please refer to Supplemental Table 3 for exact site coordinates.

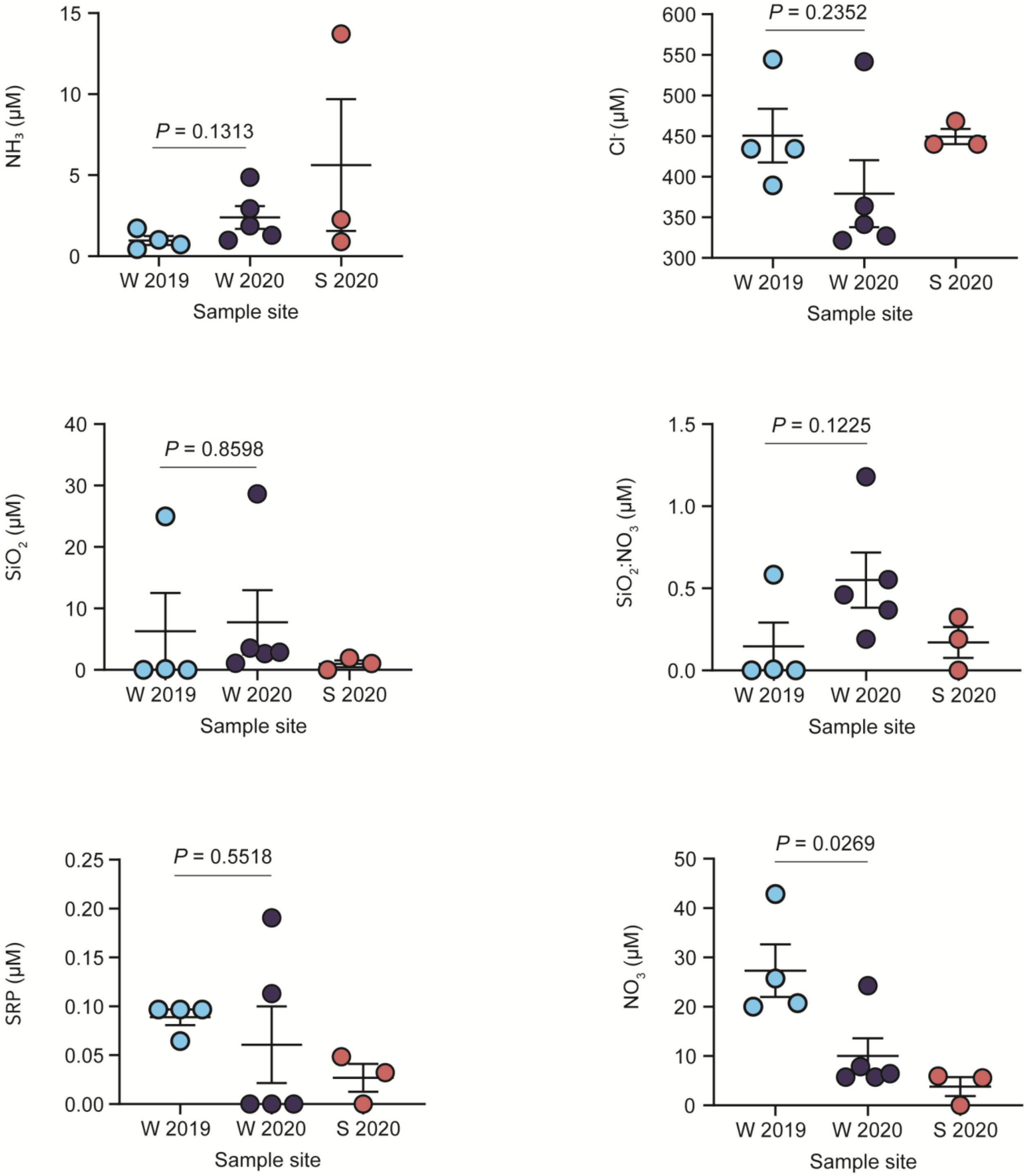

Supplemental Figure 3. Nutrient measurements (as µM) for each site. Two-tailed unpaired *t*-test results are shown for the winter 2019 (ice-covered) versus winter 2020 (ice-free) comparisons.

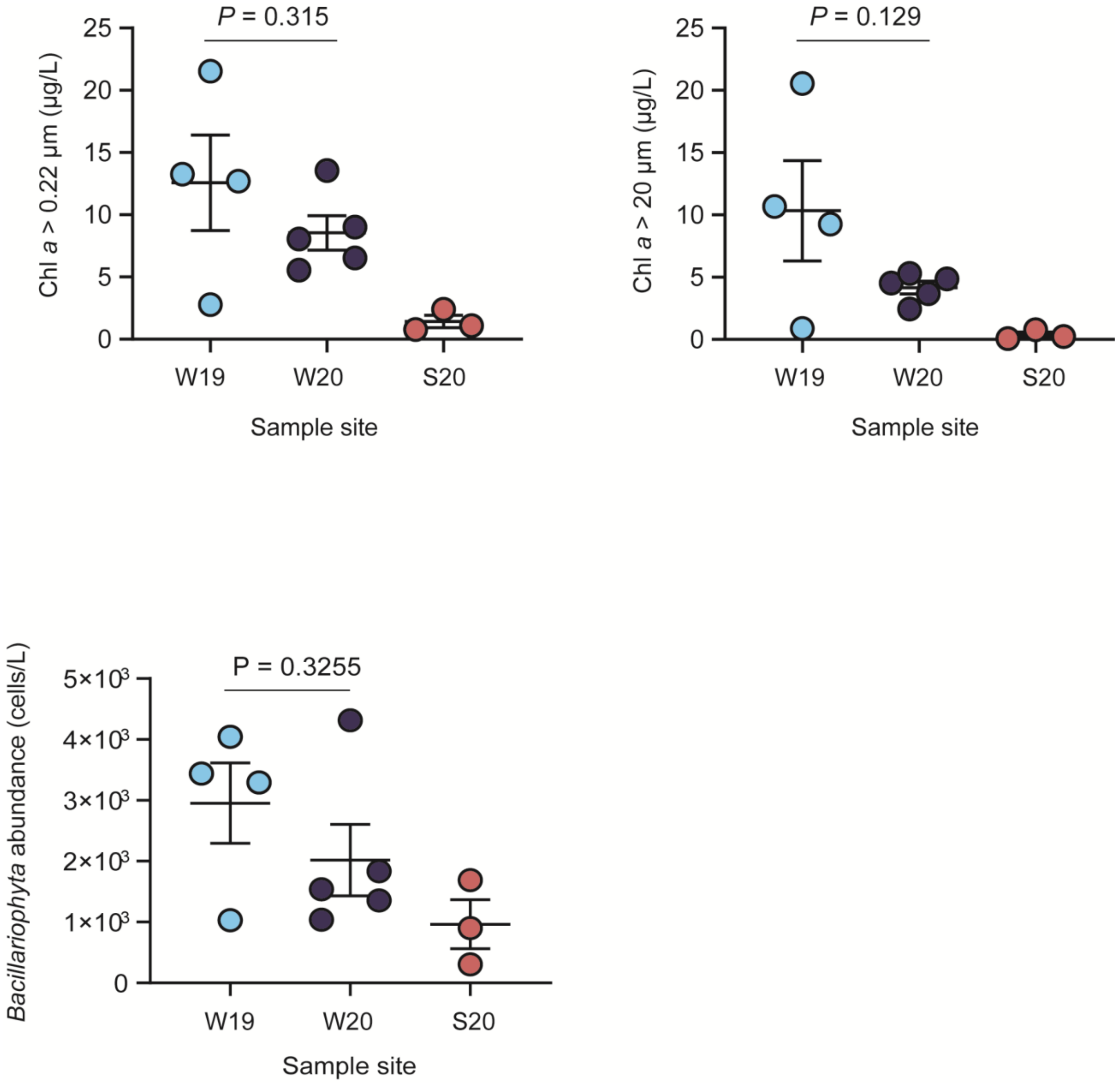

Supplemental Figure 4. Phytoplankton biomass quantified as Chl *a* (µg/L, > 0.22 and > 0.20 µm) and diatom (*Bacillariophyta*) cell counts (cells/L) at each site. Chl *a* values are shown as the mean of the biological replicates (refer to Table S5). Diatom cell counts represent the sum of *Aulacoseira islandica*, *Stephanodiscus* spp., centric diatoms 5-20 µm, *Fragilaria* spp., *Asterionella formosa*., and *Nitzschia* spp. Two-tailed unpaired *t*-test results are shown for the winter 2019 (ice-covered) versus winter 2020 (ice-free) comparisons.

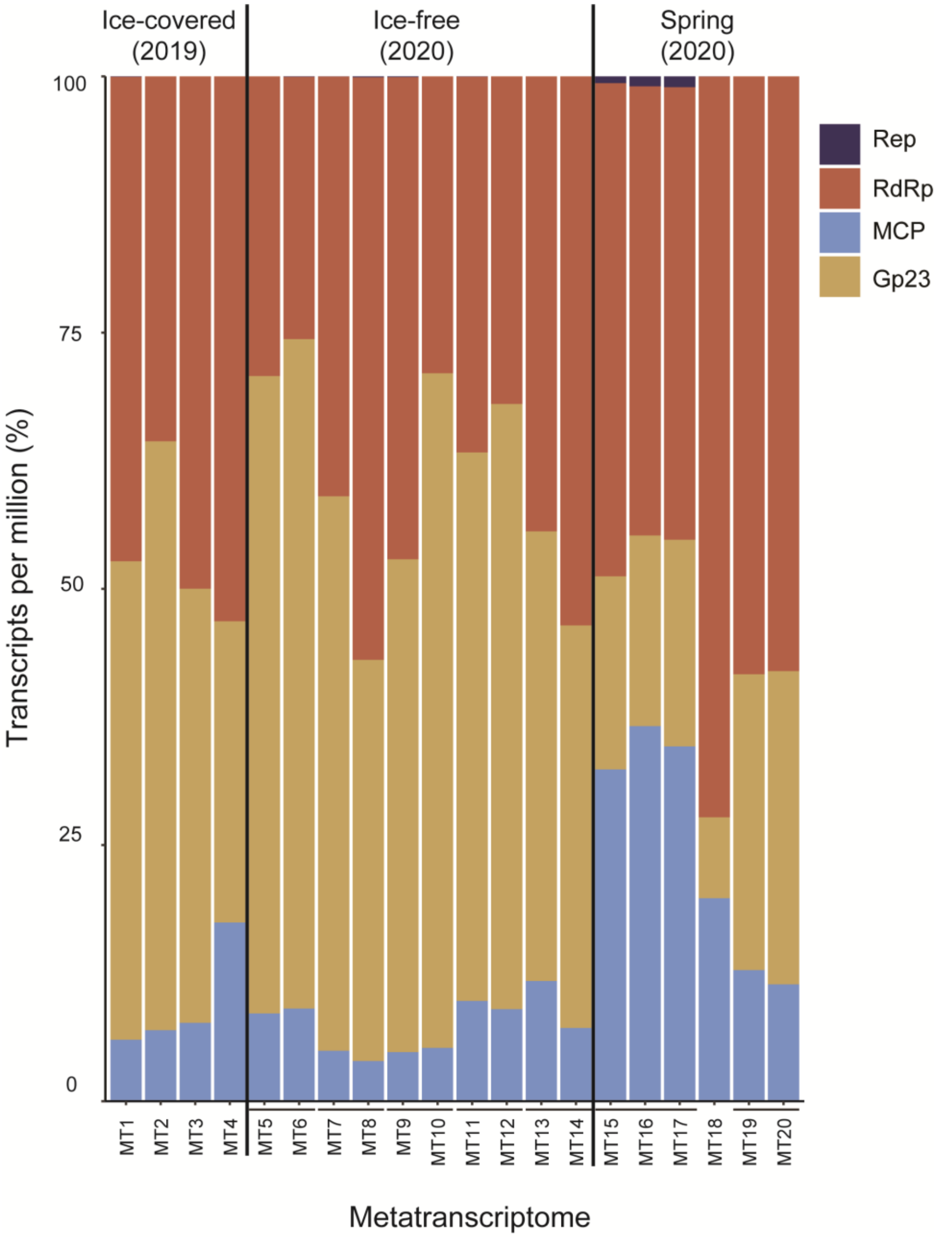

Supplemental Figure 5. Relative transcript abundance (TPM) summed by viral hallmark gene type. Biological replicates are connected by horizontal bars.

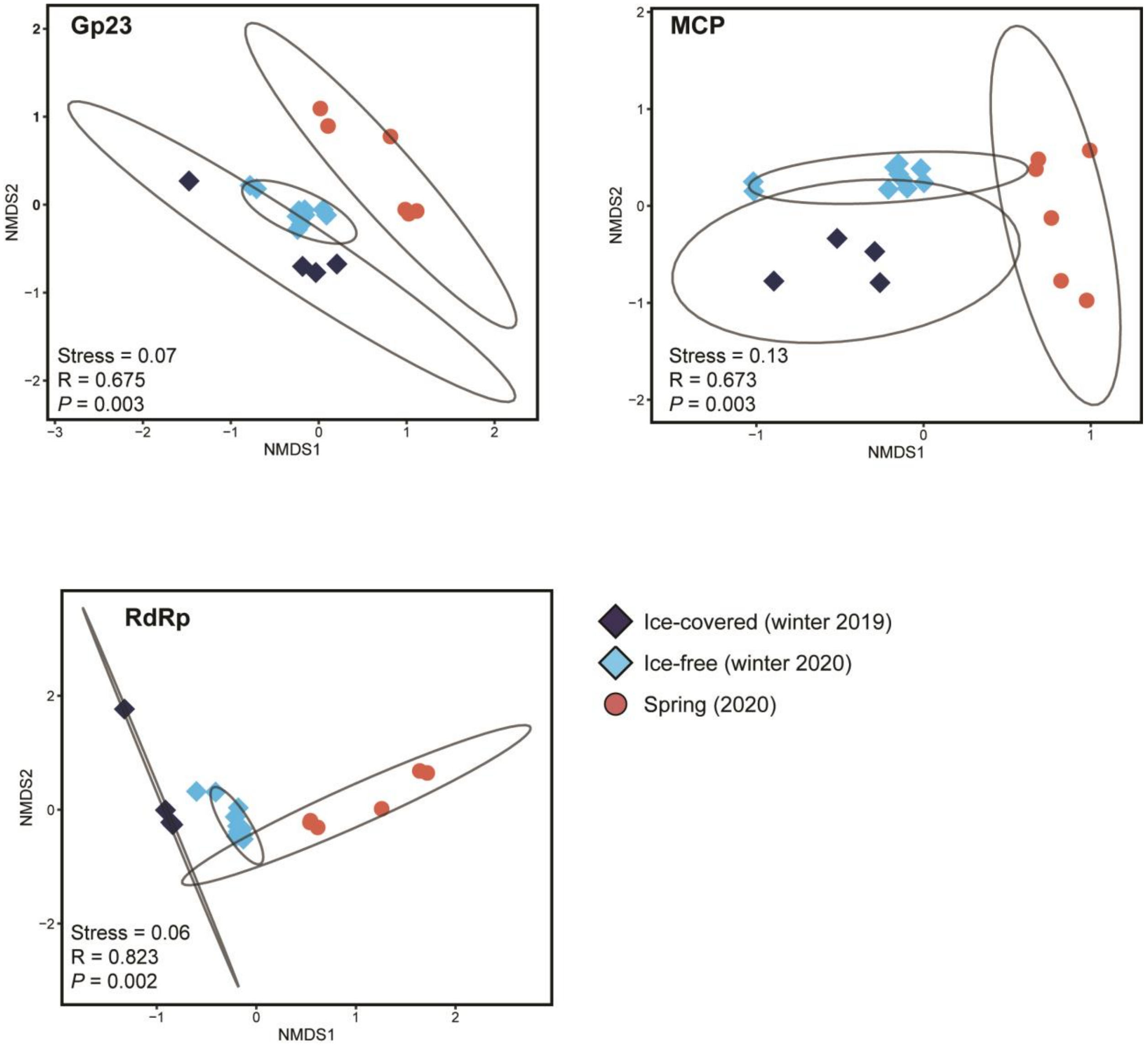

Supplemental Figure 6. nMDS plot illustrating clustering of active viral community composition by season based on relative transcript abundance (TPM) of individual viral hallmark gene types. ANOSIM test results are shown for the pairwise winter comparison. Ellipses represent 95% confidence intervals for the three seasons sampled (winter 2019, winter 2020, and spring 2020).

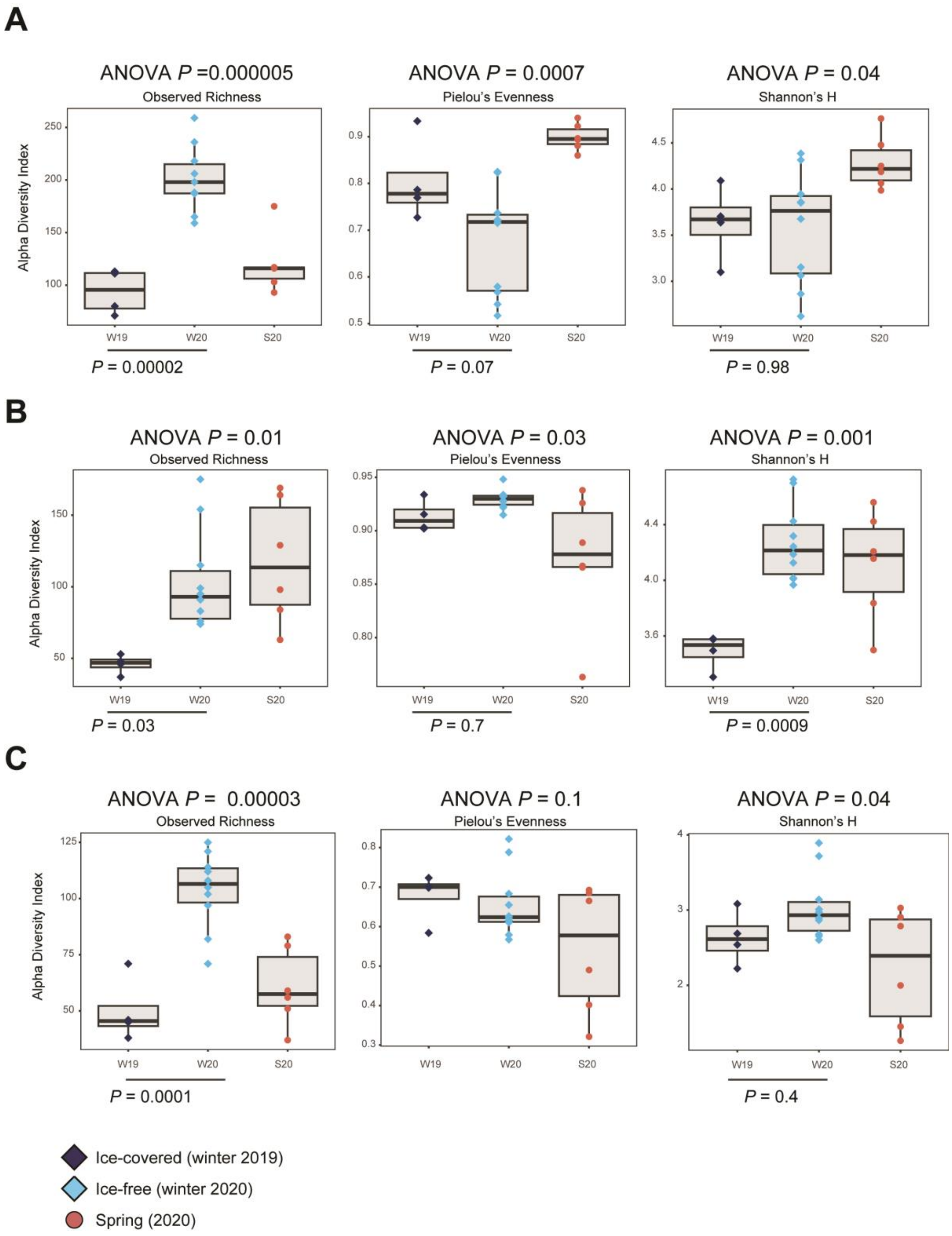

Supplemental Figure 7. Alpha diversity metrics grouped by season for the individual viral hallmark gene types **A)** Gp23, **B)** MCP, and **C)** RdRp. Tukey's HSD is shown for the ice-covered (winter 2019, W19) and ice-free (winter 2020, W20) comparison when applicable.

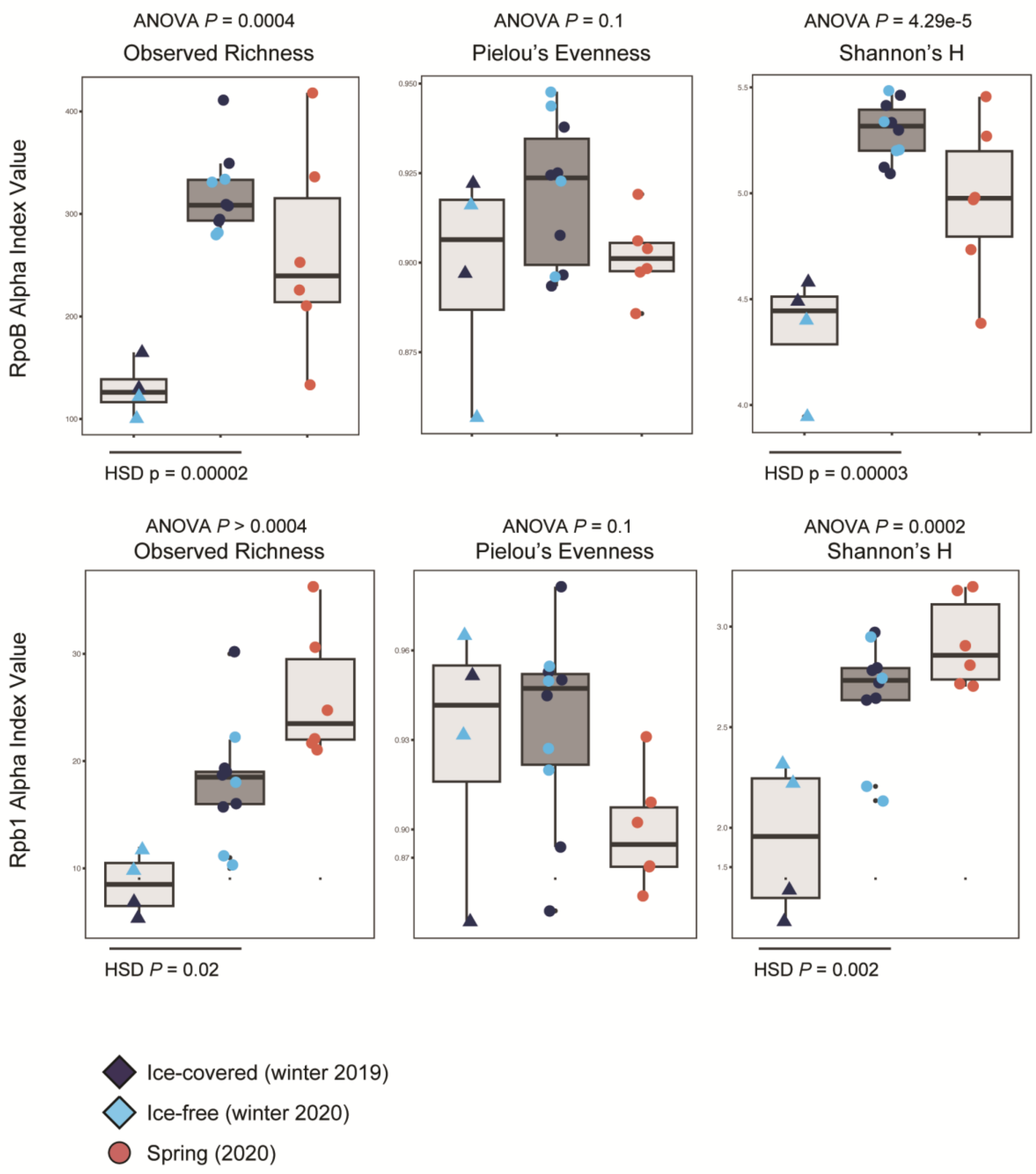

Supplemental Figure 8. Alpha diversity metrics grouped by season for RpoB (prokaryotic) and Rpb1 (eukaryotic) marker gene profiles. Tukey's HSD is shown for the ice-covered (winter 2019, W19) and ice-free (winter 2020, W20) comparison when applicable.

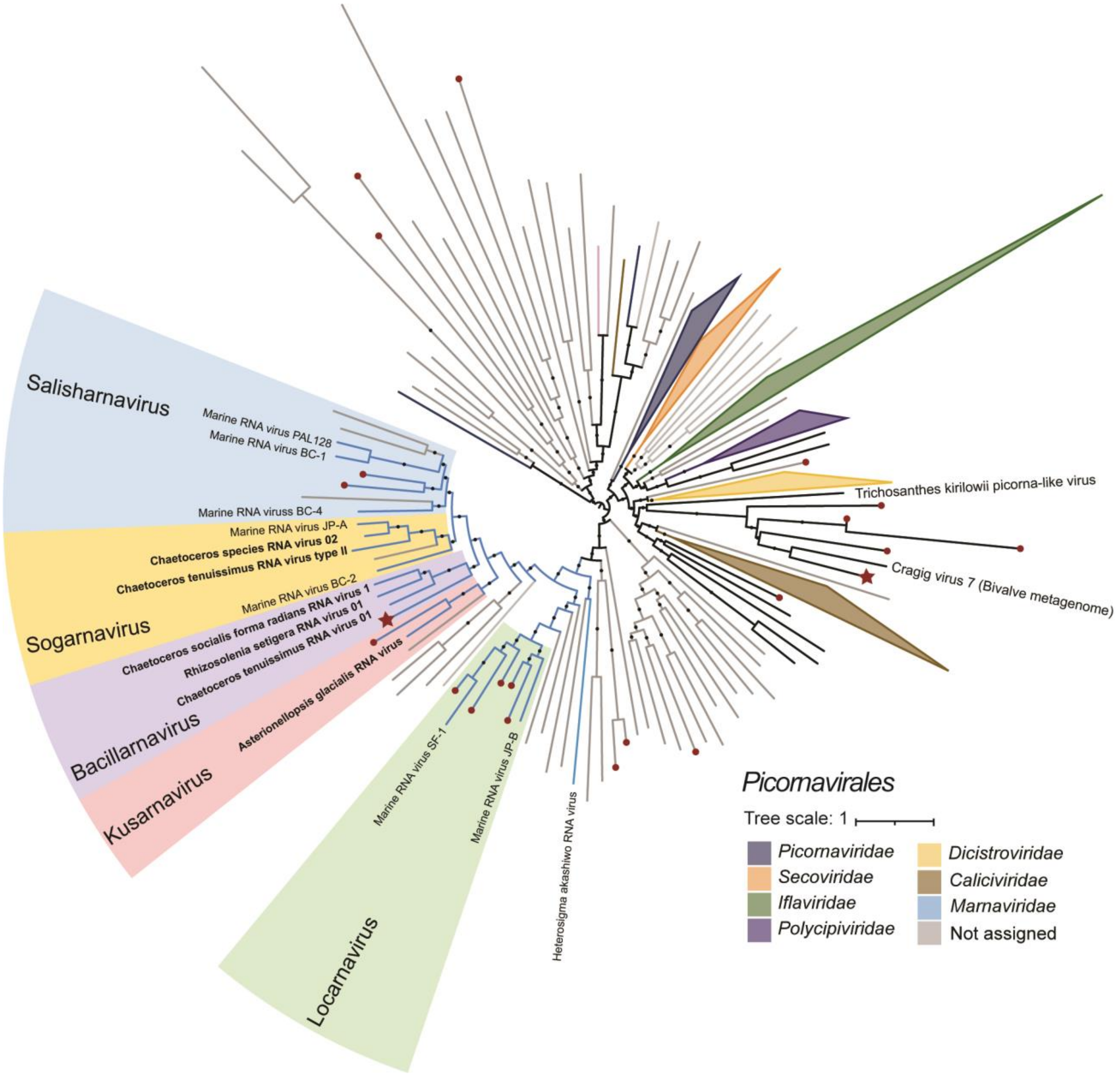

Supplemental Figure 9. Maximum-likelihood (ML) phylogenetic placement of *Picornavirales* (phylum *Pisuviricota*) RdRp hallmark genes. Nodes ending in red dots represent Lake Erie RdRp. Nodes ending in red stars represent Lake Erie RdRp with greater that 0.5% contribution to average dissimilarity between the ice-covered and ice-free sample groups.

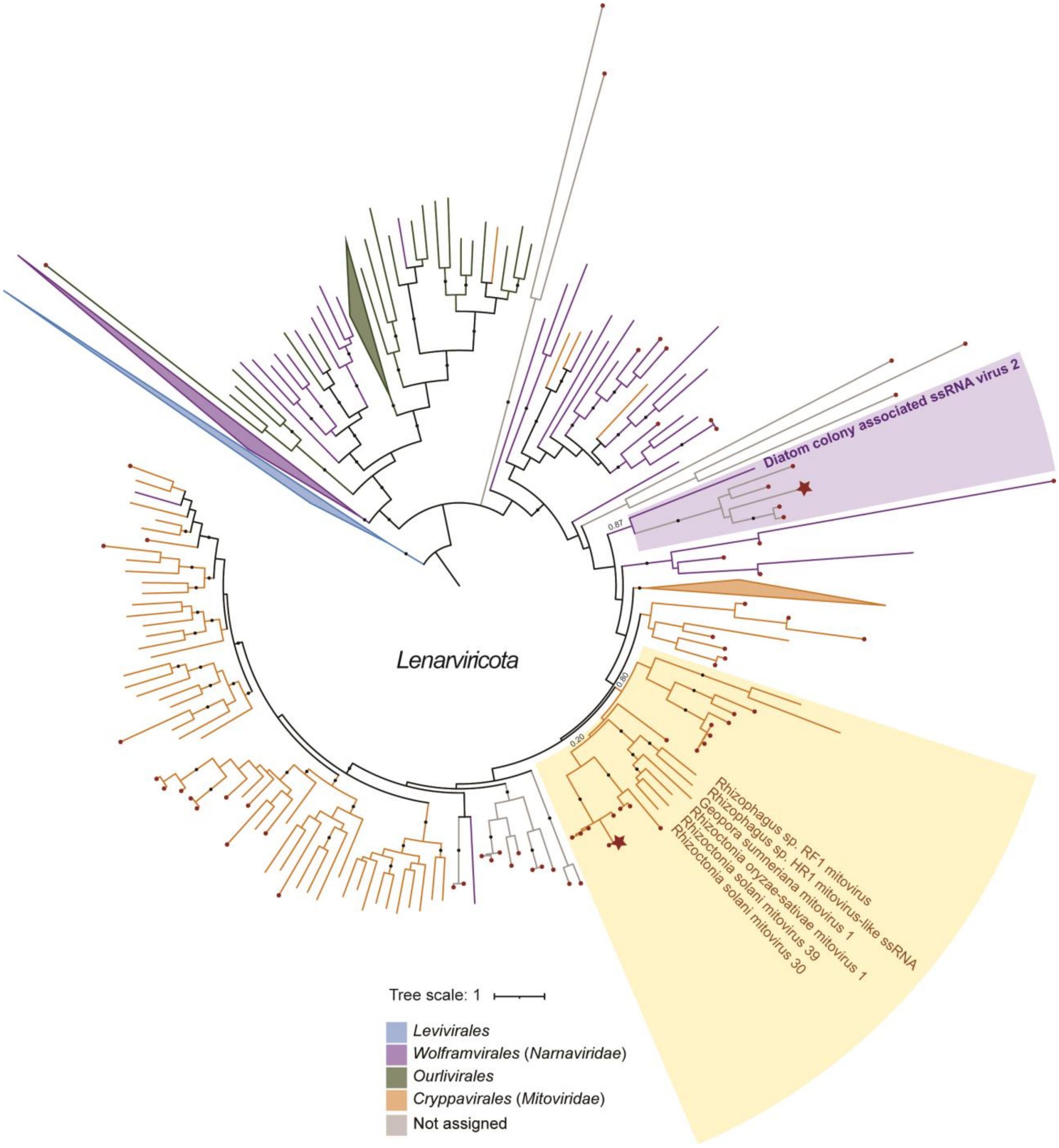

Supplemental Figure 10. ML phylogenetic placement of *Lenarviricota* RdRp hallmark genes. Nodes ending in red dots represent Lake Erie RdRp. Nodes ending in red stars represent Lake Erie RdRp with greater that 0.5% contribution to average dissimilarity between the ice-covered and ice-free sample groups.

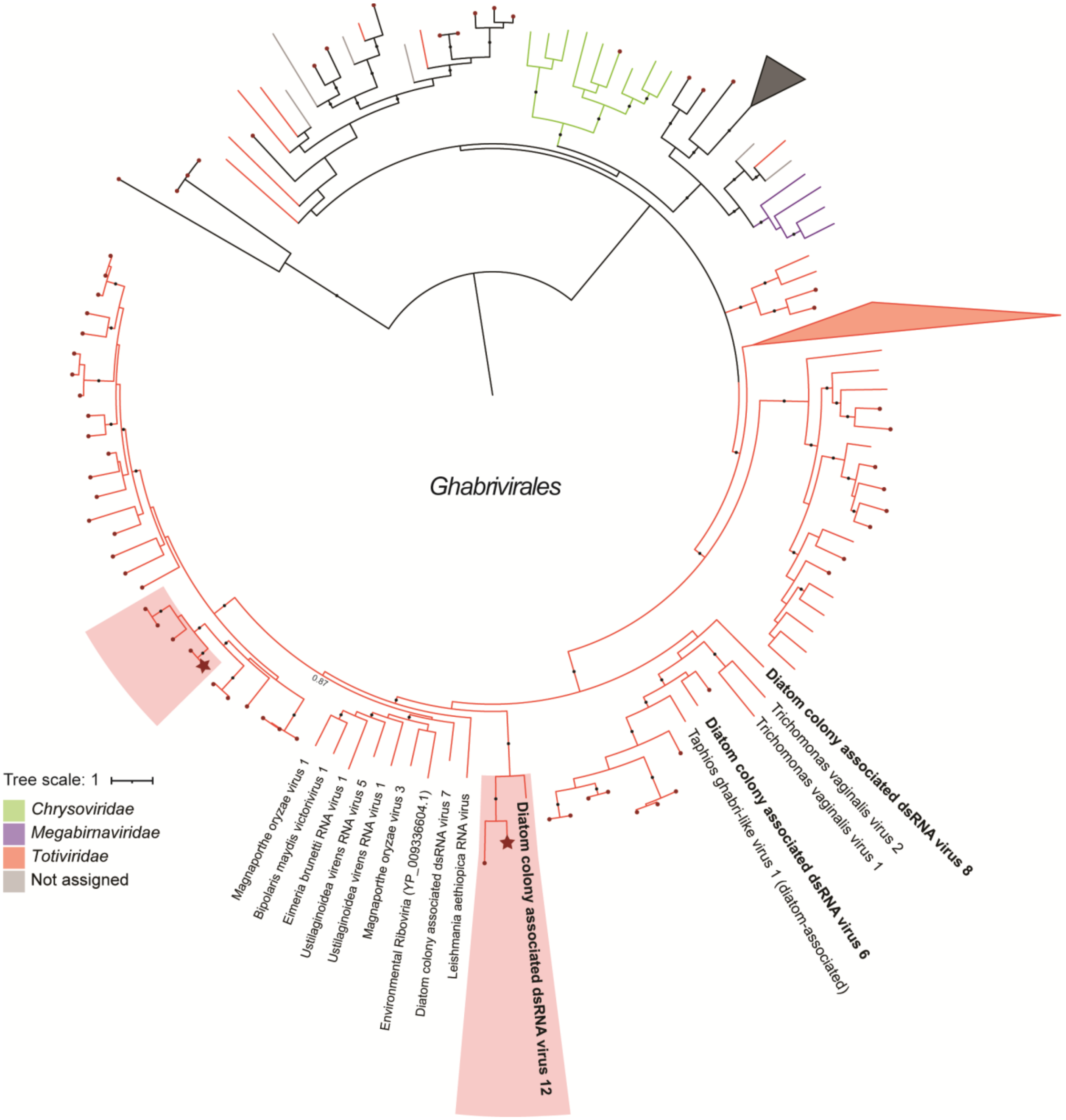

Supplemental Figure 11. ML phylogenetic placement of *Ghabrivirales* (phylum *Duplornaviricota*) RdRp hallmark genes. Nodes ending in red dots represent Lake Erie RdRp. Nodes ending in red stars represent Lake Erie RdRp with greater that 0.5% contribution to average dissimilarity between the ice-covered and ice-free sample groups.

SUPPLEMENTAL TABLES

Supplemental Table 1. Co-assembly statistics generated by QUAST v5.0.2. All QUAST statistics are based on contigs of size >= 500 bp unless otherwise noted.

# contigs (>= 0 bp)	1677102
# contigs (>= 1000 bp)	148925
# contigs (>= 5000 bp)	2139
# contigs (>= 10000 bp)	288
# contigs (>= 25000 bp)	8
# contigs (>= 50000 bp)	0
Total length (>= 0 bp)	966763097
Total length (>= 1000 bp)	247639904
Total length (>= 5000 bp)	15925927
Total length (>= 10000 bp)	4001501
Total length (>= 25000 bp)	258591
Total length (>= 50000 bp)	0
# contigs	639361
Largest contig	40227
Total length	574417227
GC (%)	44.46
N50	891
N75	641
L50	190932
L75	383548
# N's per 100 kbp	0

Supplemental Table 2. Viral hallmark genes identified in the metatranscriptome co-assembly *via* BLASTP.

**Viral Group**	**Marker**	**BLASTP Database Source**	**No. Identified**
Caudoviricetes	Gp23	PF07068	505
	Tail Sheath	PF17481, PF17482, and PF04984	239
	PolB (gp34)	PF03175 and PF00136	33
	Gp20	PF06810	1
	Integrase	PF00589	1
	Excisionase	PF06806	0
	CI Repressor	PF07022	0
	Cro Repressor	IP000655	0
	TerL	PF05876 and PF20454	1
Nucleocytoviricota	MCP	NCVOG0022	615
	PolB	NCVOG0038	9
Orthornavirae	RdRp	RdRp-Scan	397
Cressdnaviricota	Rep	Kazlauskas *et al*. and Moniruzzaman *et al*.	8

Supplemental Table 3. Sampling site locations and water conditions for the 20 whole-water samples. Site ID corresponds to the map in Figure S2. ND indicates data was not collected.

Sample ID	Collection date	Latitude	Longitude	Site ID	Ice cover (%)	Ice thickness (cm)	Snow cover (%)	Water temp (°C)	Air temp (°C)	Baro press (Hg)	Wind (kt)	Wind (°)	DO (mg/L)	DO (%)	Cond uS/cm	pH
MT1	2-26-2019	41.839333	-82.5215	S1	100	15	0	ND	-7.8	29.85	3	90	ND	ND	ND	ND
MT2	2-6-2019	41.806	-82.383833	S2	100	2.5	0	ND	-7.8	29.85	6	70	ND	ND	ND	ND
MT3	3-11-2019	41.7497	-81.8352	S3	100	8	0	ND	-1.1	29.57	6	260	ND	ND	ND	ND
MT4	3-11-2019	41.8658	-82.6407	S4	90	10	0	ND	-1.1	29.59	16	260	ND	ND	ND	ND
MT5	2-14-2020	41.82856667	-82.50111667	S5	0	0	0	0.0	-8.3	ND	ND	ND	12.3	89.1	248.0	9.4
MT6	2-14-2020	41.82856667	-82.50111667	S5	0	0	0	0.0	-8.3	ND	ND	ND	12.3	89.1	248.0	9.4
MT7	2-14-2020	41.73851667	-82.27431667	S6	0	0	0	1.89	-8.9	ND	ND	ND	12.4	89.4	240.0	9.4
MT8	2-14-2020	41.73851667	-82.27431667	S6	0	0	0	1.89	-8.9	ND	ND	ND	12.4	89.4	240.0	9.4
MT9	2-14-2020	41.66628333	-82.08163333	S7	0	0	0	2	-9.4	ND	ND	ND	12.3	88.9	254.0	9.4
MT10	2-14-2020	41.66628333	-82.08163333	S7	0	0	0	2	-9.4	ND	ND	ND	12.3	88.9	254.0	9.4
MT11	3-2-2020	41.66591667	-82.07911667	S8	0	0	0	2.22	2.8	29.07	ND	ND	ND	ND	237.0	9.96
MT12	3-2-2020	41.66591667	-82.07911667	S8	0	0	0	2.22	2.8	29.07	ND	ND	ND	ND	237.0	9.96
MT13	3-2-2020	41.77033333	-82.35166667	S9	0	0	0	1.67	2.2	29.05	ND	ND	ND	ND	255.0	9.89
MT14	3-2-2020	41.77033333	-82.35166667	S9	0	0	0	1.67	2.2	29.05	ND	ND	ND	ND	255.0	9.89
MT15	5-1-2020	42.43045	-81.20616667	S10	0	0	0	9.67	11.3	ND	14	315	ND	ND	273.0	9.13
MT16	5-1-2020	42.43045	-81.20616667	S10	0	0	0	9.67	11.3	ND	14	315	ND	ND	273.0	9.13
MT17	5-1-2020	42.43045	-81.20616667	S10	0	0	0	9.67	11.3	ND	14	315	ND	ND	273.0	9.13
MT18	5-22-2020	42.42953333	-81.20435	S11	0	0	0	9.67	11.3	ND	5.3	127	12.43	108.5	266	9.03
MT19	6-8-2020	42.2817	-81.67171667	S12	0	0	0	15.66	14.8	ND	9	140	ND	ND	266	9.03
MT20	6-8-2020	42.2817	-81.67171667	S12	0	0	0	15.66	14.8	ND	9	140	ND	ND	266	9.03

Supplemental Table 4. Dissolved (< 0.2 µm) and particulate (unfiltered) nutrient measurements for the 20 whole-water samples.

	Dissolved							Particulate	
**Sample ID**	**NH3 (mg/l)**	**CL (mg/l)**	**S04 (mg/l)**	**NO2 (mg/l)**	**NO3 (mg/l)**	**SIO2 (mg/l)**	**SRP (mg/l)**	**TP (mg/l)**	**TN (mg/l)**
MT1	0.014	15.4	19.2	0	0.36	0.01	0.003	0.0349	0.442
MT2	0.01	15.4	18.9	0	0.29	0	0.003	0.0201	0.292
MT3	0.006	19.3	18.9	0	0.28	0	0.003	0.0149	0.276
MT4	0.024	13.8	15.4	0	0.6	1.5	0.002	0.0127	0.253
MT5	0.0411	12.9	17.6	0	0.08	0.1583	0.0059	ND	0.27
MT6	0.0411	12.9	17.6	0	0.08	0.1583	0.0059	ND	0.27
MT7	0.0181	11.4	16.8	0	0.08	0.0653	0.0035	ND	0.369
MT8	0.0181	11.4	16.8	0	0.08	0.0653	0.0035	ND	0.369
MT9	0.0262	19.2	24.1	0	0.11	0.1737	0	ND	0.274
MT10	0.0262	19.2	24.1	0	0.11	0.1737	0	ND	0.274
MT11	0.0682	12.1	17	0	0.34	1.7211	0	ND	0.245
MT12	0.0682	12.1	17	0	0.34	1.7211	0	ND	0.245
MT13	0.0137	11.6	16.5	0	0.09	0.2136	0	ND	0.403
MT14	0.0137	11.6	16.5	0	0.09	0.2136	0	ND	0.403
MT15	0.1921	16.6	17.2	0.063	0	0	0.0015	0.0075	0.208
MT16	0.1921	16.6	17.2	0.063	0	0	0.0015	0.0075	0.208
MT17	0.1921	16.6	17.2	0.063	0	0	0.0015	0.0075	0.208
MT18	0.0125	15.6	16.6	0.065	0.0777	0.0635	0	0.0044	0.196
MT19	0.0316	15.6	16.8	0.062	0.083	0.115	0.001	0.0027	0.173
MT20	0.0316	15.6	16.8	0.062	0.083	0.115	0.001	0.0027	0.173

Supplemental Table 5. Phytoplankton biomass estimates (Chl *a*) and diatom taxa enumeration (shown as cell/mL).

Sample ID	Chl *a* (> 0.2 µm) (µg/L)				Chl *a* (>20 µm) (µg/L)				*Aulacoseira islandica*	*Stephanodiscus* spp.	*Fragilaria* spp.	*Nitzschia*	*Asterionella*	centric diatoms (5-20 µm)
MT1	16.47	26.58	ND	ND	18.09	23.00	ND	ND	1.44E+03	2.35E+03	0.00E+00	ND	ND	ND
MT2	12.95	13.89	13.04	13.2	8.15	10.55	12.04	11.93	9.62E+02	2.35E+03	3.60E+01	ND	ND	ND
MT3	14.23	12.45	11.41	ND	9.8	8.01	9.94	ND	7.60E+02	2.48E+03	5.40E+01	ND	ND	ND
MT4	2.87	2.24	3.23	ND	1.1	0.82	0.76	ND	1.20E+01	1.02E+03	ND	ND	ND	ND
MT5	8.18	7.76	8.23	ND	4.6	4.32	4.66	ND	3.89E+02	1.13E+03	4.00E+01	1.10E+01	4.00E+00	2.64E+02
MT6	8.18	7.76	8.23	ND	4.6	4.32	4.66	ND	3.89E+02	1.13E+03	4.00E+01	1.10E+01	4.00E+00	2.64E+02
MT7	10.96	9.15	6.94	ND	4.86	5.29	5.73	ND	3.87E+02	8.26E+02	1.49E+02	1.40E+01	1.60E+01	1.49E+02
MT8	10.96	9.15	6.94	ND	4.86	5.29	5.73	ND	3.87E+02	8.26E+02	1.49E+02	1.40E+01	1.60E+01	1.49E+02
MT9	17.43	8.95	14.27	ND	5.38	4.36	ND	ND	4.52E+02	6.28E+02	7.90E+01	1.50E+01	1.40E+01	1.70E+02
MT10	17.43	8.95	14.27	ND	5.38	4.36	ND	ND	4.52E+02	6.28E+02	7.90E+01	1.50E+01	1.40E+01	1.70E+02
MT11	6.43	7.4	5.78	ND	2.15	2.50	2.69	ND	1.50E+02	2.32E+02	5.80E+01	1.00E+01	1.70E+01	3.56E+03
MT12	6.43	7.4	5.78	ND	2.15	2.50	2.69	ND	1.50E+02	2.32E+02	5.80E+01	1.00E+01	1.70E+01	3.56E+03
MT13	5.68	5.56	5.44	ND	3.41	3.64	3.99	ND	3.85E+02	2.32E+02	2.60E+01	1.00E+00	3.10E+01	1.20E+02
MT14	5.68	5.56	5.44	ND	3.41	3.64	3.99	ND	3.85E+02	2.32E+02	2.60E+01	1.00E+00	3.10E+01	1.20E+02
MT15	2.49	2.65	2.05	ND	0.82	0.78	0.77	ND	9.60E+01	3.12E+02	4.40E+02	0	0	ND
MT16	2.49	2.65	2.05	ND	0.82	0.78	0.77	ND	9.60E+01	3.12E+02	4.40E+02	0	0	ND
MT17	2.49	2.65	2.05	ND	0.82	0.78	0.77	ND	9.60E+01	3.12E+02	4.40E+02	0	0	ND
MT18	1.24	0.82	1.23	ND	0.19	0.21	0.32	ND	0	1.90E+01	2.40E+01	1.00E+00	5.50E+01	ND
MT19	0.78	0.82	0.74	ND	0.06	0.08	0.06	ND	0	0	1.18E+02	6.00E+00	1.53E+02	ND
MT20	0.78	0.82	0.74	ND	0.06	0.08	0.06	ND	0	0	1.18E+02	6.00E+00	1.53E+02	ND

Supplemental Table 6. Taxonomic estimate based on ML phylogeny of the identified viral hallmark genes. Viral hallmark gene detection in the winter (either ice-covered or ice-free samples) versus spring only was determined via read mapping.

			**Detected in**	
	**Taxonomy estimate**	**No. hallmark genes identified**	**Winter**	**Spring only**
**Gp23**	Uncultured myophage	468	439	29
	Cyanomyophage	2	1	1
	Pelagibacter phage	6	6	0
	Uncertain	29	22	7
	**Total**	**505**	**468**	**37**
**MCP**	*Pimascovirales*	24	10	14
	Asfuvirales	9	8	1
	Pandoravirales	4	3	1
	Algavirales	18	9	9
	Imitervirales	557	395	162
	Uncertain	3	2	1
	**Total**	**615**	**427**	**188**
**RdRp**	*Pisuviricota*	92	76	16
	Duplornaviricota	99	84	15
	Lenarviricota	84	72	12
	Kitrinoviricota	53	43	10
	Negarnaviricota	17	13	4
	Birnaviridae	1	1	0
	Permutotetraviridae	1	1	0
	Uncertain	50	42	8
	**Total**	**397**	**332**	**65**

Supplemental Table 7. ANOVA results for alpha diversity metrics compared between the three seasons sampled (winter 2019, winter 2020, and spring 2020). Diversity metrics were generated from the relative transcript abundance (TPM) table of all viral hallmark genes.

	Comparison	Observed richness	Pielou's evenness	Shannon's H
**Main test**	**Season**	**2.20E-05**	0.545	0.746
	**Ice-covered/Ice-free**	**0.0000205**	NA	NA
	**Ice-free/spring**	**0.0242167**	NA	NA
**Tukey's HSD**	**Ice-covered/spring**	**0.0056772**	NA	NA

Supplemental Table 8. ANOVA results for alpha diversity metrics compared between the three seasons sampled (winter 2019, winter 2020, and spring 2020). Diversity metrics were generated from the relative transcript abundance (TPM) table of separate viral hallmark gene types.

		**Gp23**		
	**Comparison**	**Observed richness**	**Pielou's evenness**	**Shannon's H**
**Main test**	**Season**	**5.26E-06**	**7.36E-04**	**3.58E-02**
**Tukey's HSD**	**Ice-covered/Ice-free**	**1.93E-05**	7.42E-02	9.78E-01
	**Ice-free/spring**	3.50E-01	2.78E-01	1.37E-01
	**Ice-covered/spring**	**1.03E-04**	**5.91E-04**	**3.42E-02**

		**MCP**		
	**Comparison**	**Observed richness**	**Pielou's evenness**	**Shannon's H**
**Main test**	**Season**	**1.38E-02**	**2.50E-02**	**1.25E-03**
**Tukey's HSD**	**Ice-covered/Ice-free**	**3.12E-02**	7.27E-01	**9.04E-04**
	**Ice-free/spring**	**1.37E-02**	2.27E-01	**1.17E-02**
	**Ice-covered/spring**	7.12E-01	**1.96E-02**	5.63E-01

		**RdRp**		
	**Comparison**	**Observed richness**	**Pielou's evenness**	**Shannon's H**
**Main test**	**Season**	**2.73E-05**	1.09E-01	**3.53E-02**
**Tukey's HSD**	**Ice-covered/Ice-free**	**1.16E-04**	NA	4.34E-01
	**Ice-free/spring**	5.80E-01	NA	5.18E-01
	**Ice-covered/spring**	**3.03E-04**	NA	**2.89E-02**

Supplemental Table 9. Additional Spearman correlations between various richness and relative abundance metrics. Significant correlations are bolded and in grey. Relative abundance inputs were either the summed TPM within a library for all genes (i.e., all genes annotated as prokaryotic or *Bacillariophyta*) or for a specific marker gene (e.g., genes annotated as RpoB).

		**Spearman**	
**Comparison**		**rho**	**p**
***Bacillariophyta* TPM**	**Prokaryotic TPM**	**-0.8329670**	**0.0002166**
***Bacillariophyta* TPM**	**RpoB TPM**	**-0.7098901**	**0.004451**
Bacillariophyta TPM	RpoB richness	-0.2615385	0.3664
Bacillariophyta TPM	Rpb1 richness	-0.0044249	0.988
Bacillariophyta TPM	RpoB TPM	0.1868132	0.5225
Bacillariophyta TPM	Rpb1 TPM	-0.2615385	0.3664
***Bacillariophyta* TPM**	**Viral hallmark gene TPM**	**-0.8153846**	**0.0003791**
**Prokaryotic TPM**	**Viral hallmark gene richness**	**0.9384615**	**6.86E-07**
***Bacillariophyta* TPM**	**Viral hallmark gene richness**	**-0.8153846**	**0.0003791**
***Bacillariophyta* TPM**	**Gp23 TPM**	**-0.7318681**	**0.002925**
Bacillariophyta TPM	MCP TPM	-0.1340659	0.6477
***Bacillariophyta* TPM**	**RdRp TPM**	**-0.8769231**	**3.83E-05**
Bacillariophyta TPM	Viral hallmark gene richness	-0.1956044	0.5028
Bacillariophyta TPM	Gp23 richness	-0.1870188	0.522
Bacillariophyta TPM	MCP richness	-0.1584159	0.5886
***Bacillariophyta* TPM**	**RdRp richness**	**-0.6072611**	**0.02127**
Prokaryotic TPM	Gp23 richness	0.4576460	0.09988
**Prokaryotic TPM**	**MCP richness**	**0.5808584**	**0.02939**
**Prokaryotic TPM**	**RdRp richness**	**0.6226626**	**0.01739**
**RpoB/Rpb1 richness**	**Viral hallmark gene richness**	**0.9340659**	**1.028E-06**
**RpoB richness**	**Gp23 richness**	**0.9636970**	**3.06E-08**
**RpoB richness**	**MCP richness**	**0.7876792**	**8.23E-04**
**RpoB richness**	**RdRp richness**	**0.6600664**	**1.02E-02**
**Rpb1 richness**	**MCP richness**	**0.7497346**	**0.002018**
Rpb1 richness	RdRp richness	0.4507267	0.1058
**Rpb1 richness**	**Gp23 richness**	**0.8815196**	**3.08E-05**

Supplemental Table 10. Select output from similarity percentage (SIMPER) analysis. Viral hallmark genes contributing to the top ~10% cumulative dissimilarity between the ice-covered and ice-free winter are shown (see Figure 4). The abundance values shown are based on the square root transformed TPM table.

		**Ice-covered (2019)**	**Ice-free (2020)**				
**Gene ID**	**Viral marker**	**Av. Abund.**	**Av. Abund.**	**Av. Diss.**	**Diss/SD**	**Contrib. (%)**	**Cumul. (%)**
gene_458515	RdRp	1.94	22.95	1.46	2.25	2.06	2.06
gene_293376	Gp23	8.46	24.46	1.2	1.65	1.7	3.76
gene_678216	Gp23	4.87	15.75	0.81	1.71	1.14	4.9
gene_1385399	RdRp	0	9.83	0.68	3.12	0.97	5.86
gene_26037	RdRp	9.23	0.87	0.62	1.07	0.87	6.73
gene_1263456	RdRp	2.68	8.71	0.43	2.3	0.61	7.35
gene_430917	RdRp	10.14	13.89	0.41	1.19	0.58	7.93
gene_1656192	Gp23	5.26	10.08	0.41	1.64	0.58	8.51
gene_626655	Gp23	0	5.56	0.39	2.7	0.56	9.06
gene_1534634	RdRp	6.68	2.38	0.38	1.25	0.53	9.6
gene_118358	Gp23	8.14	3.73	0.37	1.81	0.52	10.12
